# How far neuroscience is from understanding brains

**DOI:** 10.3389/fnsys.2023.1147896

**Published:** 2023-10-05

**Authors:** Per E. Roland

**Affiliations:** Department of Neuroscience, University of Copenhagen, Copenhagen, Denmark

**Keywords:** understanding brains, neuroscience concepts, spatial brain dynamics, intrinsic activity, spontaneous ongoing activity, brain mechanisms, dendrites, axons

## Abstract

The cellular biology of brains is relatively well-understood, but neuroscientists have not yet generated a theory explaining how brains work. Explanations of how neurons collectively operate to produce what brains can do are tentative and incomplete. Without prior assumptions about the brain mechanisms, I attempt here to identify major obstacles to progress in neuroscientific understanding of brains and central nervous systems. Most of the obstacles to our understanding are conceptual. Neuroscience lacks concepts and models rooted in experimental results explaining how neurons interact at all scales. The cerebral cortex is thought to control awake activities, which contrasts with recent experimental results. There is ambiguity distinguishing task-related brain activities from spontaneous activities and organized intrinsic activities. Brains are regarded as driven by external and internal stimuli in contrast to their considerable autonomy. Experimental results are explained by sensory inputs, behavior, and psychological concepts. Time and space are regarded as mutually independent variables for spiking, post-synaptic events, and other measured variables, in contrast to experimental results. Dynamical systems theory and models describing evolution of variables with time as the independent variable are insufficient to account for central nervous system activities. Spatial dynamics may be a practical solution. The general hypothesis that measurements of changes in fundamental brain variables, action potentials, transmitter releases, post-synaptic transmembrane currents, etc., propagating in central nervous systems reveal how they work, carries no additional assumptions. Combinations of current techniques could reveal many aspects of spatial dynamics of spiking, post-synaptic processing, and plasticity in insects and rodents to start with. But problems defining baseline and reference conditions hinder interpretations of the results. Furthermore, the facts that pooling and averaging of data destroy their underlying dynamics imply that single-trial designs and statistics are necessary.

## 1. Introduction

Understanding how a system works, usually means to understand the mechanisms by which its elements interact. If the major interaction mechanisms are known and ideally described mathematically, one has a theory of the system. So, the reason why neuroscientists do not understand how brains and central nervous systems work is that there is no theory of brains and central nervous systems. A scientific theory of a central nervous system (CNS) is an experimentally based general set of explanations of how the elements in a CNS interact at all scales of observation, i.e., from the molecular to the macroscopic scale. At the molecular scale neuroscience is guided by the theory of molecular biology. Although molecular neuroscience does not have a mathematical framework, it identifies molecules, provides rules explaining genetic replication, transcription, synthesis, interactions, and transformation of organic molecules. However, at the cellular, and especially supracellular scales of observation, neuroscience is far from having a guiding theory.

The purpose of this article is to identify why it is so difficult to build a theory of brains and point to domains where neuroscience seems stuck in that process. Indeed, experimental neuroscience produce a rapidly increasing number of results. Based on the current structure of (systems) neuroscience, I will argue, it is impossible to put all results together to a theory of a CNS. The reasons are not primarily lack of experimental data, nor lack of methods. So, those who expect a review of how far neuroscience has reached and expect to find a list of what we do not yet know, please stop reading here. Rather the reasons for lack of progress are obstacles inherent in current neuroscientific practice which hinder us from knowing more about brains.

In this paper I use a theory of science approach to locate weaknesses in neuroscientific practices.

**Figure 1:**
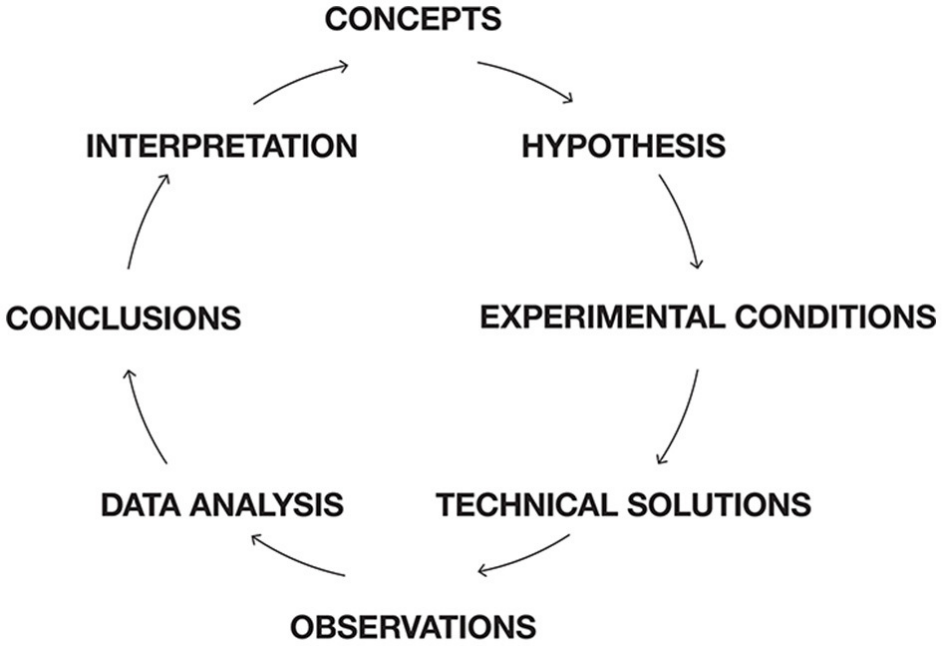
Scientific scheme for neuroscience. Roadmap for this paper. Instead of having theory on top, neuroscience have a set of concepts guiding hypothesis formation. Most of the obstacles for progress are conceptual. Conceptual glitches propagate to hypotheses, creations of experimental conditions, data analysis, and interpretations of results. First, concepts, which cannot efficiently relate to brain activities are identified. Then obstacles for models of brain functions based on brain structure and assumptions of connectivity are exposed. It is shown that cognitive tasks are not localized to specific sets of cortical areas. Unchartered issues and obstacles in understanding dendritic processing in single neurons and populations of neurons are discussed. Difficulties of distinguishing task related bran activities from spontaneous and intrinsic activities are discussed and so is the relation between autonomous and stimulus driven brain activities. The assumption that time is the independent variable for brain activities is analyzed and experimental results incompatible with this hypothesis are presented. Dynamic systems theory and models are blind to spatial interactions, limiting this approach. These obstacles are followed by suggestions to overcome them. Technically, experimental neuroscience is mostly challenged by revealing fast processes at the single neuron scale and limited by difficulties of including primates. Experimental practice neglects difficulties of finding true reference conditions, neglects the problematic assumptions that experimental animals always are naïve, and trials are statistically independent. Similarly, data are analyzed by bandwidth filters, temporal and spatial averaging removing important aspects of brain mechanisms. Finally, avoiding these many obstacles could make it easier to reliably interpret experimental results.

Neuroscience works, as other scientific disciplines, with a scientific scheme (Figure 1). Normally theory would be at the top in Figure 1. However, in the absence of a guiding theory, neuroscientists form hypotheses guided by concepts. If a concept used in neuroscience does not match brain activities, neuroscience will not progress in that direction. This is the danger of not having a theory in which relations among concepts are defined without inconsistencies. Figure 1 may serve as a roadmap for this paper, dealing with obstacles in the neuroscientific process.

Within the realms in Figure 1, one can identify obstacles of progress. The obstacles of progress indirectly identify frontiers in (systems) neuroscience. In many cases, it is possible to give suggestions that could circumvent an obstacle, push it, or eliminate it. In this effort, I build on results provided by many wise colleagues during workshops aimed to understand how brains and central nervous systems work (see Acknowledgments). This article, however, is my personal extract.

## 2. Conceptual obstacles

### 2.1. Lack of neuroscientific concepts

Anyone studying neuroscience and reading textbooks and neuroscientific literature gets introduced to the concepts that neuroscientists use to explain how central nervous systems are anatomically constructed and how neurons work. Some concepts are rooted in reproducible experimental results from neuroscience itself: synapse, transmitter release, membrane currents, action potentials, ion-channels, excitation, inhibition, etc. Some concepts are more loosely used: top-down, bottom-up, dorsal and ventral streams, parallel processing, or recurrent processing with reference to anatomical schemes of connectivity.

**Figure 2:**
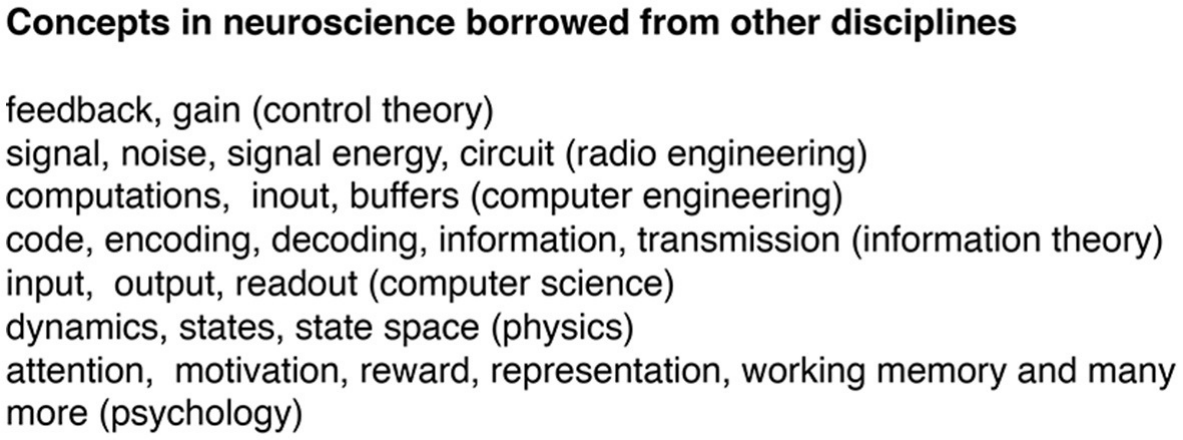
Examples of concepts in neuroscience borrowed from other disciplines. These concepts are analogies explaining how other systems work. In neuroscience, these concepts are attempts to explain how brains work by explaining how other non-brain systems work. Analogies cannot explain brain mechanisms because they lack ontological connection to measurable brain variables. In other words, it is obscure how the concepts relate to brain variables. To remedy this, neuroscientists sometimes make new definitions of the concept. For example, gain gets re-defined as the relative increase in spike rate for a neuron. In other instances, raw data get transformed to comply with borrowed concepts. For example, oscillations are rare in *in vivo* measurements. The irregular field potentials and EEG recordings then gets filtered to produce band limited oscillations (see further under Experimental obstacles and data analysis). In short, the use of borrowed concepts implies unnecessary troubles and uncertainties in the whole neuroscientific process (Figure 1).

Many concepts, however, are borrowed from other scientific disciplines (Figure 2). The concepts shown in Figure 2 are used to explain how the systems in their mother disciplines work technically and (often) mathematically. These borrowed concepts are used as analogies in neuroscience. But the borrowed concepts are not tailored to explain (more complex) biological systems such as brains. Logically, analogies cannot and do not explain how neurons collaborate to achieve the whole repertoire of CNS activities. Psychological concepts have been a rich source for importing brain functions into neuroscience. Psychological concepts are made to explain and link human behavior to particular social or environmental conditions, but not fitted to explain the mechanisms by which neurons produce this behavior.

Recently, dynamics and tools from dynamical systems theory are used to characterize the collective activities of neurons (see later). The analogies shown in Figure 2 are also used as assumptions, as part of scientific hypotheses, and to interpret experimental results. If we remove all analogies and metaphors as attempts to explain brain mechanisms in neuroscience, will we lose understanding of brains? Logically, the answer is no. But one may claim that brains have certain properties which could be labeled by psychological concepts. For example, brains can show attention. In this case, which is not the rule, it is possible to hypothesize and experimentally identify physiological mechanisms creating a pre-stimulus activity making it possible to detect, say near threshold stimuli (see later). When this is experimentally supported, it would be scientifically efficient to refer to this brain mechanism, rather than referring to a psychological concept with unclear ontological connection to brains. This replacement gives a precise definition that can be experimentally tested. Neuroscience should explore all possible conditions with no conceptual restrictions (see later). When we abandon the analogies, neuroscientists would be forced to analytically form concepts and hypotheses of brain mechanisms based on experimental results. Lack of concepts explaining collective interactions of neurons at all spatial scales of observation is a real obstacle for neuroscience.

*Conceptual frontier 1: Develop concepts strongly rooted in experimental results explaining how neurons (and glia) interact at all scales*.

### 2.2. Brain structure and models

**Figure 3:**
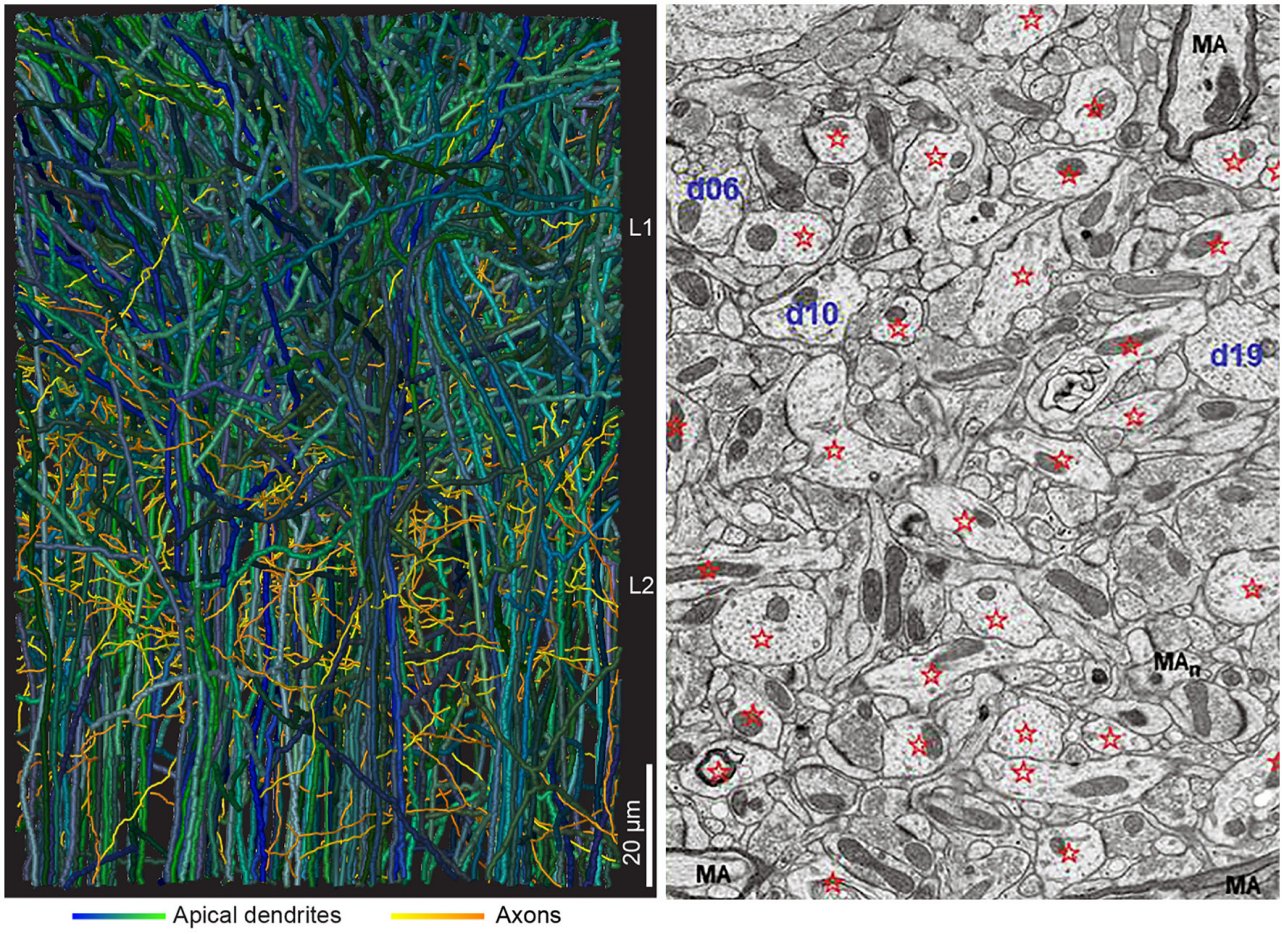
Interdigitating dendrites. **(Left)** Two hundred thirteen reconstructed apical dendrites from layer 2 (61 gray dendrites) and layers 3, 4, and 5 (152 dendrites) from mouse anterior cingulate cortex (from Karimi et al., 2020, with permission). In the volume, ~2,000 dendrites from adjacent neurons and multiple axonal branches from adjacent local and distal neurons will complete the picture. **(Right)** Electron microscopic image, 10 × 12 μm, from adult rat CA1 stratum radiatum, with dendrites identified by stars and d (number). MA, myelinated axon (from Harris et al., 2022, with permission).

Connectomics produce reconstructions showing the challenging microstructure of cortical networks (Figure 3). The challenge is to extract the functionally most relevant connectivity to build models of CNS activities. An alternative is to simulate the whole connectome. Currently insect (*Drosophila*) and mammalian connectomes available are partial connectomes showing synaptic connections of only a part of the CNS (Scheffer et al., 2020). So, in practice, simulations still evolve in a local network (for example Markram et al., 2016; Schmidt et al., 2018). Apart from the trouble of building the model, the model must also be validated against experimental results, which would be quite an undertaking.

So far CNS models have no lasting eigen activity. There are some relatively detailed models of cerebral cortex (Izhikevich and Edelmann, 2008; Kumar et al., 2008; Eliasmith et al., 2012; Markram et al., 2016; Schmidt et al., 2018). These models are started by injecting noise, stimuli, or Poisson spike trains. However, when the afferent stimulation ceases, the spiking activity dies out. Mammalian brains, and most likely also insect and zebrafish CNS, have eigen activity as ever-changing ongoing spiking and membrane currents no matter whether they are stimulated or not, awake or at sleep (Rudolph et al., 2007; Yap et al., 2017; Stringer et al., 2019; Davis et al., 2020; Marques et al., 2020; McCormick et al., 2020; Siegle et al., 2021; Willumsen et al., 2022).

*Conceptual frontier 2: Build a brain model with modifiable, but everlasting ongoing changes of membrane potentials and spiking like that in mammalian brains*.

### 2.3. Functions and CNS activities

Except in mathematics, the word function assumes activity to fulfill a purpose or obtain a goal. Following the line of thinking in the lack of concepts section, one ought to be careful reading purposes or psychology into CNS activities (Buzsaki, 2020). A more neutral description is CNS activities. CNS activities can be measured directly as changes of trans-membrane currents (which includes action potentials), transmitter release and binding, receptor induced biochemical changes, synthesis of brain specific proteins and other compounds, activity of transmembrane pumps and transporters. CNS activities can be measured indirectly as field potentials, changes in magnetic fields (see technical obstacles). What people and animals experience, think, memorize, and how they behave, as a general hypothesis, are consequences of CNS activities at many scales. Arriving at a full description transcending all scales of observation it the task of neuroscience. This task meets further obstacles.

#### 2.3.1. Are CNS activities carried out by separate loops, circuits, modules, or one large network?

**Figure 4:**
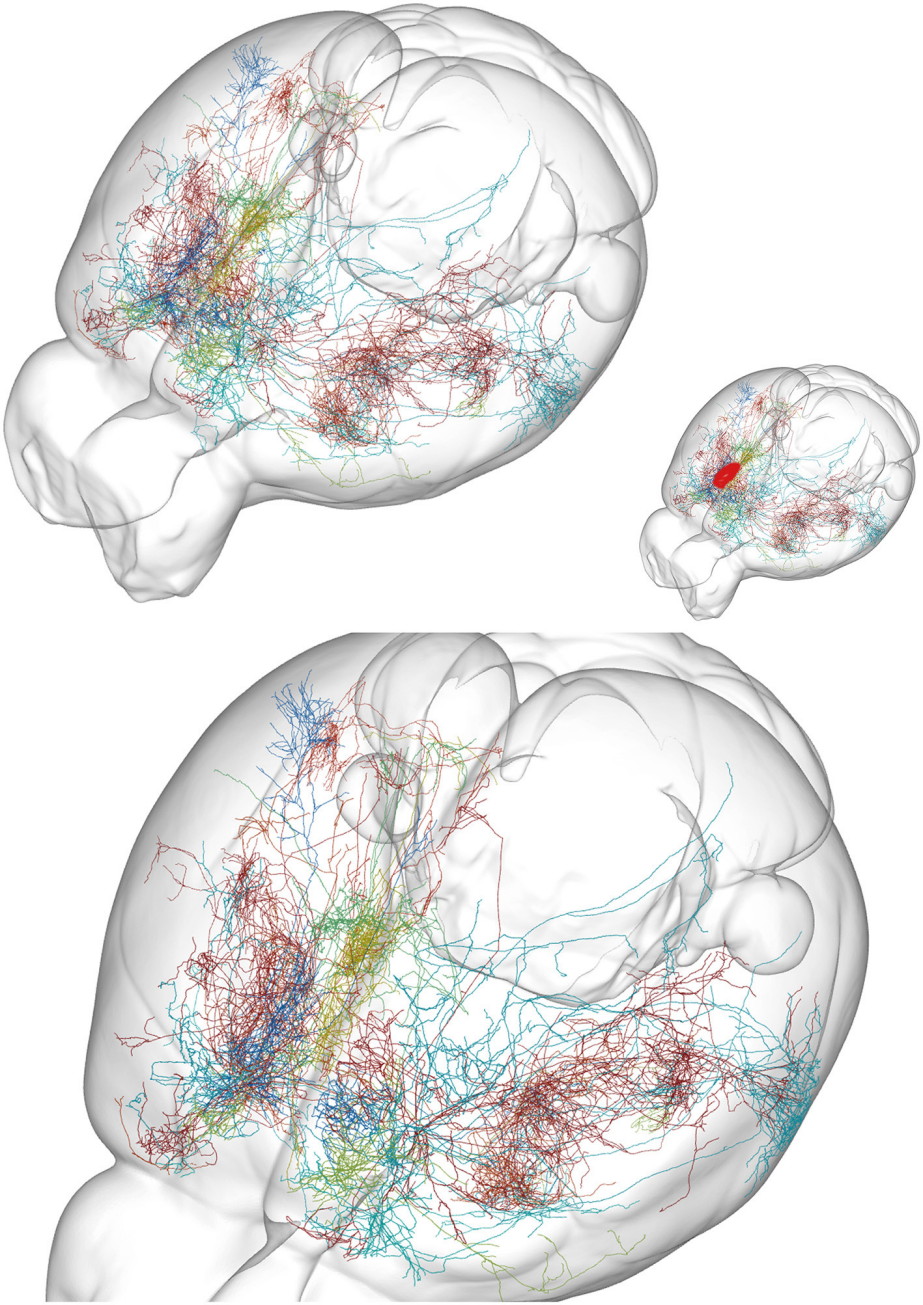
Examples of axon anatomy. Ten axons targeting prelimbic area in the mouse. The prelimbic area is small, located at the rostral and mesial surface of the frontal lobe (approximate location red in insert). **(Top)** Overview of the mouse brain. **(Bottom)** Close view. Each axon targeting the area branches at successive positions to produce an exponentially increasing number of axonal branches. An axon can have 1,000 branches (Wu et al., 2014). A single action potential (AP) in the initial part of such an axon then at each branch point give rise to two APs, one traveling in each branch. With no failures (Alcami and El Hady, 2019) this gives around 500 action potentials traveling in the roughly 500 terminal branches. Although several branches of one axon target the prelimbic area, many of its branches also end in several other cortical areas. From the MouseLight database, http://mlneuronbrowser.janelia.org. Axons belong to the following single neurons in series AA: 0138, 0241, 0344, 0397, 0402, 0802, 0842, 0883, 0897, and 1425. Four axons originate from motor cortex layer 2/3, two from motor cortex layer 5, one from adjacent anterior cingulate cortex, one from visual association area AM, one from ventral anterior nucleus of thalamus, and one from the intralaminar rhomboid nucleus of thalamus. The finest axonal branches (Figure 3) are not visible with this method.

The ideas that chains of neurons (sometimes organized in cortical-subcortical loops), micro-circuits, and modular organized cortical columns are responsible for brain activities have been criticized. The reasons were unrealistic simplifications of the actual synaptic connectivity neglecting actual dendritic and axonal anatomy (Figures 3, 4). These ideas also neglect divergence of connections to other structures than the members of the loops, micro-circuits, or columns (Alito and Usrey, 2005; Rockland, 2010, 2021; Foster et al., 2021; Shepherd and Yamawaki, 2021).

**Figure 5:**
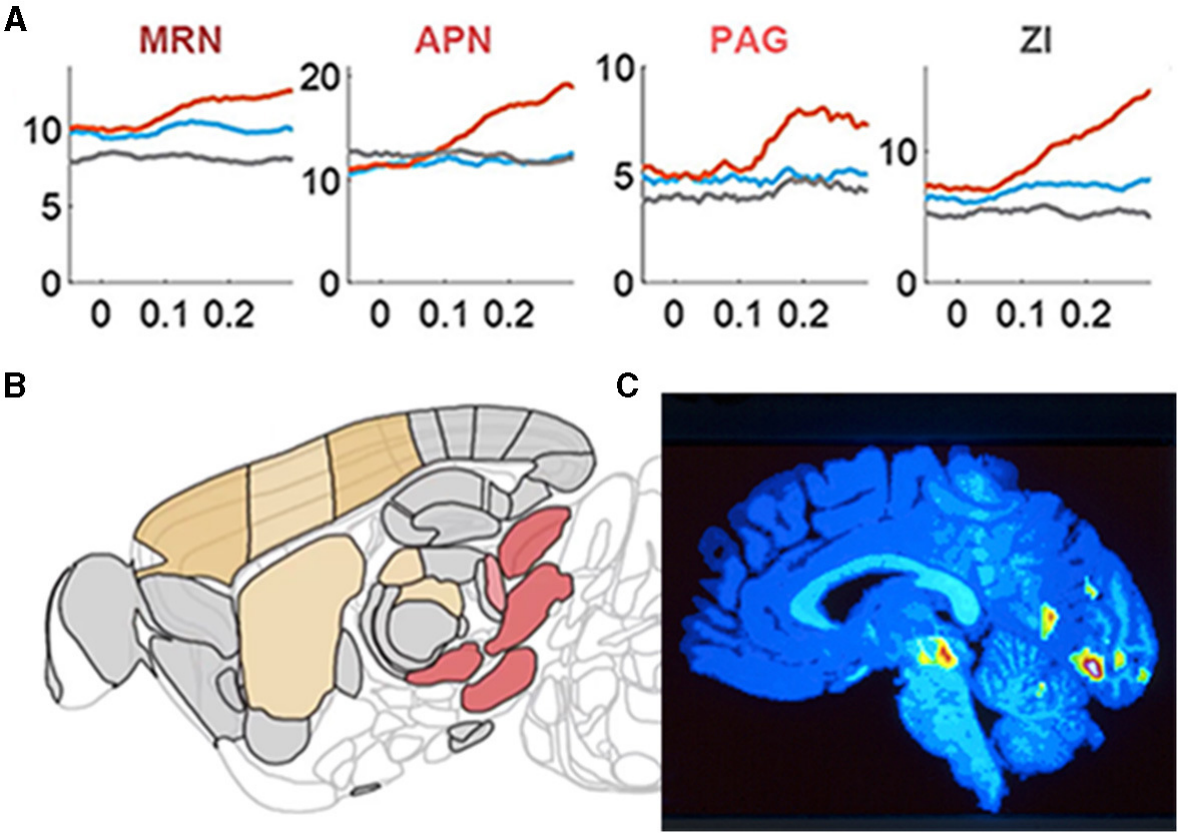
Brain stem nuclei participate in cognitive tasks. **(A)** *Y*-axis: population mean firing rates in task go trials (orange), task missed trials (blue), and passive sensory stimulation (gray). *X*-axis time 0 s stimulus onset/ target onset that the mice must bring into the center of the field of view. Note the different pre-stimulus rates in the midbrain reticular nucleus (MRN) and the zona incerta (ZI) and how these nuclei and the anterior pretectal nucleus (APN) and peri-aqueductal gray matter (PAG) become engaged in the action selection. **(B)** Sagittal section of the mouse brain showing these nuclei (red-brown) in the right brain stem specifically engaged in the right motor response (action selection; adapted from Steinmetz et al., 2019) with permission. **(C)** Sagittal section of the human brain showing the right side of the brain stem when normal subjects with their right thumb or right index finger respond to a faint increase in a visual or somatosensory stimulus, respectively. The color-coded significant increases in regional cerebral blood flow are located in the right midbrain reticular nucleus (and in the visual cortex; adapted from Kinomura et al., 1996) with permission.

Studies of cortical neurons operating *in vivo* show widely spreading depolarizations, excitations, and spiking. These results leave no support for activity restricted to a circumscribable location, to a specialized microcircuit or to columns (see following sections). Rather the spreading mechanisms may relate to the actual neuron anatomy with interdigitating multiple dendritic and axonal branches (Figures 3, 4). In a CNS perspective, large populations of neurons spike in many areas of cortex, sectors of basal ganglia, thalamus, other parts of the diencephalon, brain stem nuclei, cerebellum, and spinal cord, even during simpler tasks (Steinmetz et al., 2019; Wagner et al., 2019; Li and Mrsic-Flogel, 2020; Peters et al., 2021; Grün et al., 2022). Moreover, diencephalic and mesencephalic nuclei contribute significantly to choices and specific behaviors, showing that brain activities are results of interacting brain stem, cerebellar, basal ganglia, thalamic, and cortical networks (Figure 5).

*Conceptual frontier 3: Rather the crucial issue is whether the whole CNS is active, and if not, which (biophysical) mechanisms determine how far depolarizations and spiking spread in CNS?*

### 2.4. Single neuron activities

#### 2.4.1. Action potentials are for interaction: the bulk of processing in neurons take place in the dendrites

**Figure 6:**
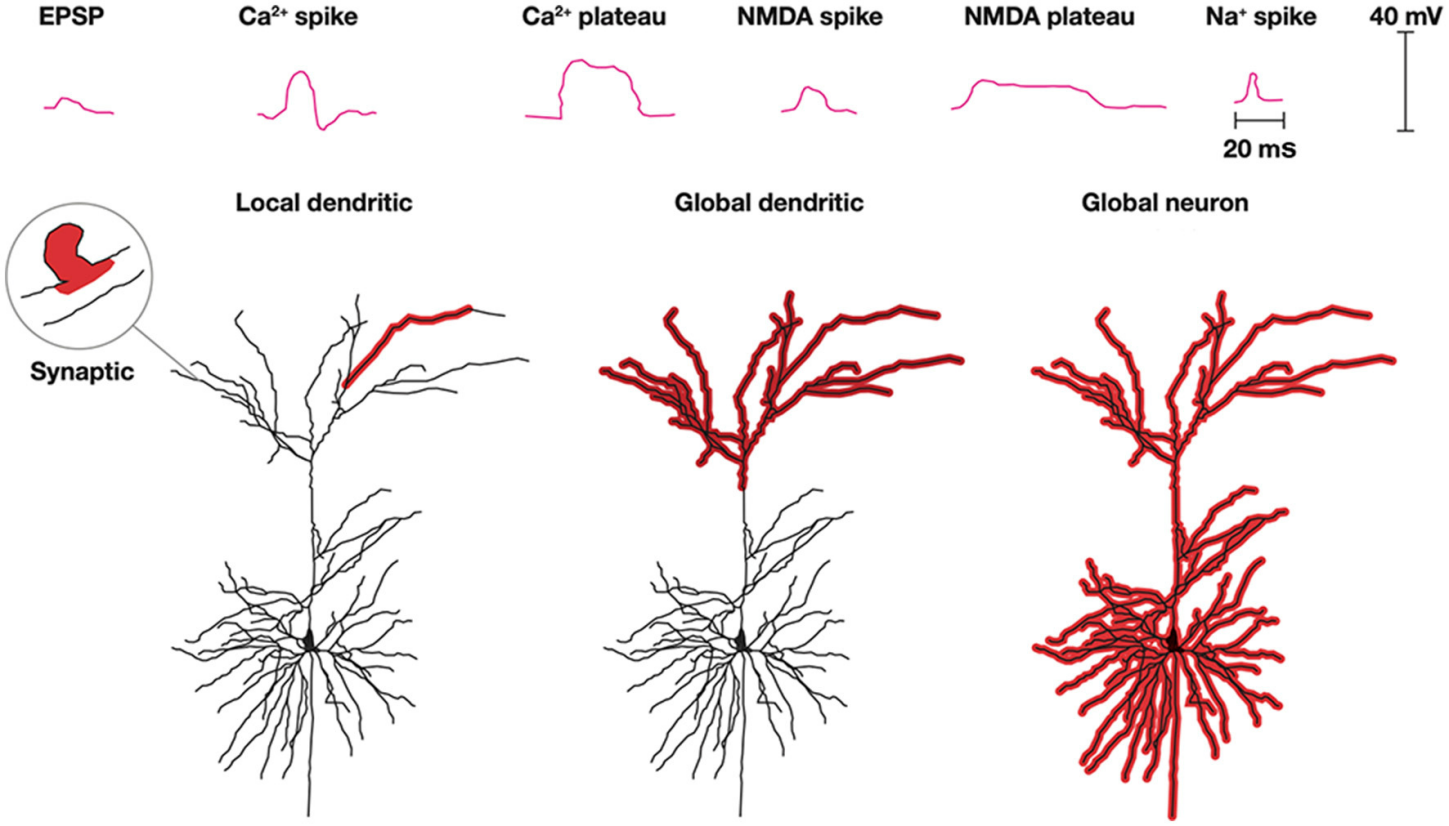
Dendritic processing. Post-synaptic processing can be an EPSP localized to a single synapse and a small part adjacent dendrite. Na^+^, NMDA, and Ca^2+^ spikes and NMDA, and Ca^2+^ plateau potentials with limited progress depolarize one or a few dendrites. Multiple spikes and plateau potentials with larger spatial progress depolarize all apical (shown) or all basal dendrites (not shown) or globally excite all dendrites and the soma (Modified from Stuyt et al., 2022) with permission.

As axons only conduct action potentials, the post-synaptic current transformations, processing, and plasticity in a neuron takes place in its dendrites (and in soma constituting the smaller part). Processing of synaptic excitatory post-synaptic potentials (EPSPs) in dendrites is complex (Figure 6). Roughly, excitatory transmitters elicit a localized EPSP in the post-synaptic spine, spreading only sparsely into the local dendrite. However, synaptic EPSPs, close in space and time, may open Ca^2+^ channels and NMDA channels in the dendrites to produce Ca^2+^ spikes or Ca^2+^ plateau potentials and NMDA spikes or NMDA plateau potentials. These spikes and plateau potentials can propagate locally in one or a few adjacent dendrites without propagating to the soma and generate action potentials (Larkum et al., 2022; Moore et al., 2022; Stuyt et al., 2022). Depending on the spatial interactions, the plateau potentials or larger spikes can also propagate to the soma and elicit an action potential (Otor et al., 2022).

Another scenario is that synaptic EPSPs close in space and time to distal dendrites may produce Ca^2+^ plateau potentials or NMDA spikes in many or all apical dendrites. Alternatively, this can happen in basal dendrites. Neither of these processes may lead to any action potentials, but nevertheless induce or restore plasticity in the active dendrites (d'Aquin et al., 2022). Similarly, apical or basal dendrites, at least in pyramidal excitatory neurons, may stay globally depolarized for up to a few seconds without this leading to a spike (Larkum et al., 2022; Stuyt et al., 2022). In addition to the Ca^2+^ and NMDA spikes, dendrites can also produce smaller Na^+^ spikes (spikelets) locally in the dendrites without this leading to action potentials (Goetz et al., 2021).

Propagation of dendritic spikes and plateau potentials to the soma often induce action potentials (Larkum et al., 2022; Moore et al., 2022; Stuyt et al., 2022). The combination of apical-somatic plateau potentials and action potentials may elicit a back-propagating action potential to many or all apical or basal dendrites. This is a mechanism that is also likely to induce or modify the plasticity of the dendrites.

The single (pyramidal) neuron can support several processes in parallel with or without spiking. Consequently, an action potential could be the result of many different dendritic processes.

*Conceptual frontier 4: Understand the local and global in vivo processing in dendrites of single neurons and their consequences for emission or withholding action potentials. This also addresses the question of which processing leads to the spike emitted*.

With rare exceptions (Mel, 1993; Jones and Kording, 2022) dendritic processing is an important fact that is neglected in models of CNS networks (Shepherd and Grillner, 2018).

### 2.5. Larger scale network activities

#### 2.5.1. Spontaneous and task-related activity

During an experimental task, e.g., 40% of the neurons in the brain and mesencephalon may increase their spiking, and up to 20% of neurons decrease their spiking, whereas the remaining 40% of the neurons do not change their ongoing spiking (Steinmetz et al., 2019; Siegle et al., 2021). However, a large proportion of neurons (up to 40% of all neurons) may not spike at all (Shoham et al., 2006; Barth and Poulet, 2012; Wohrer et al., 2013). These non-spiking neurons could also participate in the task, for example by depolarizing or hyperpolarizing their dendrites (Roland et al., 2006, 2017; Mohajerani et al., 2010; Esteves et al., 2021; Liang et al., 2021). In the future, it might be possible to estimate the proportion of neurons participating in a task in mammals by changing their transmembrane currents (see technical obstacles). For spiking, the above results might be illustrative. Thus, there are task related activities, but most studies report many spiking neurons seemingly unrelated to tasks (Urai et al., 2022). In the literature this is often called spontaneous activity.

**Figure 7:**
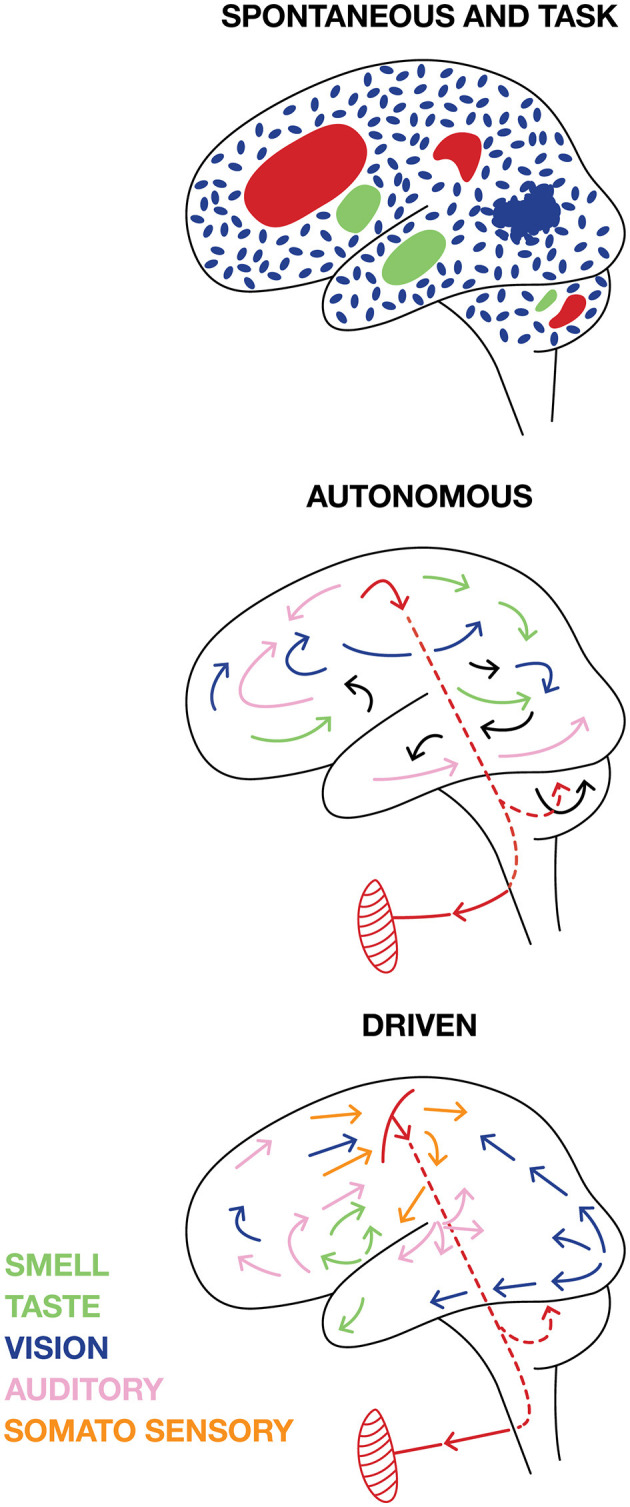
Cartoon illustrating different views on brain activities. SPONTANEOUS activities are independent of external signals and TASK activities. Spontaneous brain activity can be (blue) fluctuating irregular “background” activity spatially independent at scales < 1 mm^3^ when the brain is awake, but idle and not producing any motor activity. In other parts of the brain, INTRINSIC cognitive activities (green) not leading to any behavior engaging the network in several parts from the microscopic to macroscopic scales may co-exist with the TASK activity (red). AUTONOMOUS. The brain could be autonomous with self-organized intrinsic activities engaging the network at all scales that external stimuli and demands cannot change, but only slightly modify. The autonomous brain self-organizes motor behavior (symbolically pictured as a muscle). DRIVEN. Task related activity and external sensory stimuli and internal stimuli from the body drive brains away from spontaneous activity into sensory and cognitive activities at all scales, which eventually result in some motor behavior.

The usual distinction is between task related activity and “spontaneous ongoing activity,' i.e., CNS activities that may co-exist, but are unrelated to task and task behavior. This distinction must be made for any of the activity variables measured (spiking, synaptic, postsynaptic activity variables as defined in section 3). In practice the distinction is often set by sorting the neurons in two groups. One group for which changes in measured variables correlate with parameters of the task. The other group for which this is not so. This strategy may overlook neurons which are necessary for solving the tasks but unrelated to the stimulation and behavioral parameters (see later). The spontaneous activity may be seemingly random fluctuations of the measured variables in space and time. For example, the continuous local spatial and temporal irregular changes from slight excitation to slight inhibition prior to the stimulation as in Supplementary Video 1. This CNS activity is easy to distinguish from task CNS activity. However, during the experiment there may be neurons supporting intrinsic (cognitive) CNS activities un-related to the task (Figure 7). Separating task related activity from such “spontaneous” or more precisely self-organized intrinsic cognitive activity is difficult and may only be possible under assumptions. For example, two tasks depending on activities engaging the same part of the CNS network interfere and cannot be performed simultaneously (Herath et al., 2001) (Figure 7).

*Conceptual frontier 5: Separate self-organized intrinsic activity in CNS from task dependent activity*.

This may require examination of the whole CNS (Figure 5). Larger scale CNS activities may also be classified according to their causes. The questions raised in this section are all related to how brains and a central nervous systems self-organize their activities.

#### 2.5.2. Are brains driven or autonomous?

Until recently, neuroscience has been mainly oriented to explain how changes in the surrounds and behavioral conditions change transmembrane currents (including action potentials) and synaptic efficacy in brain neurons. Recently, there is accumulating evidence contesting this view that spiking and post-synaptic dynamics in brains are predominantly externally driven (Figure 7) (Millner, 1999; Fried et al., 2011; Buzsaki, 2019; Steinmetz et al., 2019; Cowley et al., 2020; Marques et al., 2020; Clancy and Mrsic-Flogel, 2021; Grün et al., 2022). The alternative is self-organized intrinsic activities. *Intrinsic* activity is independent of external stimuli, internal stimuli, demands and tasks, which also distinguish it from CNS activities related to bodily internal functions such as thirst, hunger, and sexual desire.

Brains are not in direct contact with the surroundings. Strictly, all spikes generated in a central nervous system are intrinsically generated. Brains can self-organize their everchanging intrinsic activity to generate slow waves, spindles, sharp wave ripples, faster irregular membrane fluctuations, dreams, and, in awake conditions, thoughts, plans, strategies, overt behavior, and (some brains) language (Figure 7). Even in primary visual and auditory cortical areas, only 5%−15% of the spikes carry information about the surround (Richmond and Optican, 1990; Heller et al., 1995; Olshausen and Field, 2006; Keyser et al., 2010; Urai et al., 2022). Similarly, the correlation of spike trains with external visual stimuli is low, typically around 0.1 in the primary visual cortex (Eriksson et al., 2010). These results are well known and indicate that 85%−95% of the spikes in a brain are autonomous. A recent large-scale study showed that external stimuli and various experimental conditions could modify fluctuations in the (multidimensional) human cortical field potential, but not perturb the underlying dynamics generating the fluctuations (Willumsen et al., 2022).

*Conceptual frontier 6: describe and classify CNS activities by how they engage the CNS network by changing CNS activities (defined in section 3). (Referring to sensory input, behavior, and psychological concepts may have limited explanatory power)*.

On the other hand, in awake conditions, focused attention and exclusion or suppression of own (intrinsic) activities can entrain field potentials partly or globally over the cerebral cortex. For example, in humans and other primates exposed to rhythmic visual or auditory stimuli, each stimulus produces a single time-locked oscillation. These time-locked oscillations can spread, with different lags, to cover the whole cortex (Besle et al., 2011; Gomez-Ramirez et al., 2011; Spaak et al., 2014; Merchant and Averbeck, 2017; Willumsen et al., 2022). Also, unexpected stimuli may elicit spreading excitation and spiking globally over cortical areas (Ferezou et al., 2007; Salkoff et al., 2020). Thus, under such circumstances, cortical networks are largely externally driven.

Most likely, brains have a certain degree of autonomy. In addition, brains regulate their sensitivity to external sensory impact. Autonomy may be distributed over different CNS structures and be differentially regulated in each structure. Even respiratory inspiration can be voluntarily modulated. Similarly, in subjects planning a motor effort, the motor system can increase the heart rate and blood pressure in advance of the motor action (Pfurtscheller et al., 2013).

*Conceptual frontier 7: Measure regulation of CNS autonomy*.

#### 2.5.3. How does intrinsic activity in brains emerge?

*Conceptual frontier 8: Find principles for how intrinsic activity in brains emerge*.

*Drosophila* and zebrafish larvae possess neurons (P1 neurons and dorsal raphe neurons, respectively) which by increased spiking mobilize several structures to produce complex behavior lasting minutes. The number of neurons triggering these behaviors is less than 100 (Jung et al., 2020; Marques et al., 2020). Details of how the trigger neurons recruit a large part of the CNS are still lacking. The changes in spiking and recruitment of many populations of neurons are examples of an intrinsically organized activity spreading to large parts of a CNS.

From mammals, there are examples of how the spiking of one or very few neurons can change the behavior and performance of an animal (Romo et al., 1998; Houweling and Brecht, 2008). However, in these examples, the animals were engaged in a task; therefore, they do not qualify as intrinsic activity (see also the text later). But the fundamental questions are still pending. For example, how many neurons are required to generate intrinsic dynamics? How many neurons are required to generate intrinsic dynamics leading to overt behavior? Dreaming is yet another example of intrinsic brain activity. How dreams start is unknown, i.e., how changes in spiking and transmembrane currents organize to produce dreams.

*Conceptual frontier 9: Reveal how changes in crucial variables (membrane potentials, transmembrane currents, and spiking) evolve to encompass larger populations of neurons in multiple structures of the CNS*.

### 2.6. Is time an independent variable for CNS operations?

An independent variable is a variable that does not depend on other variables. Time is invented by humans. Time is composed of equal units that add linearly. Time is an independent variable in Newtonian physics, but in the theory of relativity and quantum mechanics, time is not an independent variable (Rovelli, 2018). Time in neuroscience is usually regarded as an independent variable for fundamental brain processes. As external observers, scientists can timestamp every spike. Similarly, one can create mathematical functions of other measured fundamental (dependent) variables, potentials, transmembrane currents, transmitter releases, and plasticity variables using time as the exclusive independent variable. From a scientific point of view, the question is whether time is the only independent variable for operations in neurons and for CNS processes.

*Conceptual frontier 10: Examine if time is an independent variable for any activity of neurons and brains*.

#### 2.6.1. Experimental results incompatible with time as independent variable in brain activities

Spike trains have traditionally been analyzed with time as the independent variable. This could be a list of the times spikes are emitted from neurons according to an external (computer) clock or transforming the spike train to a continuous rate function of time. However, claiming that all activities in brains all evolve according to external clock time only (i.e., with time as the independent variable) is a strong hypothesis that can be proven wrong. Regarding spike trains as temporal codes carrying information to be decoded by the brain is assuming that this type of brain activity depends on time as the independent variable (Figure 2). Decades have been spent to find temporal patterns carrying the code (Barlow, 1961; Bialeck et al., 1997; Rao and Ballard, 1999; Dayan and Abbott, 2001; Bassett et al., 2020). Also simultaneously recorded neurons have been analyzed for synchrony (Gray and Singer, 1989; Abeles, 1991; Singer et al., 2019).

**Figure 8:**
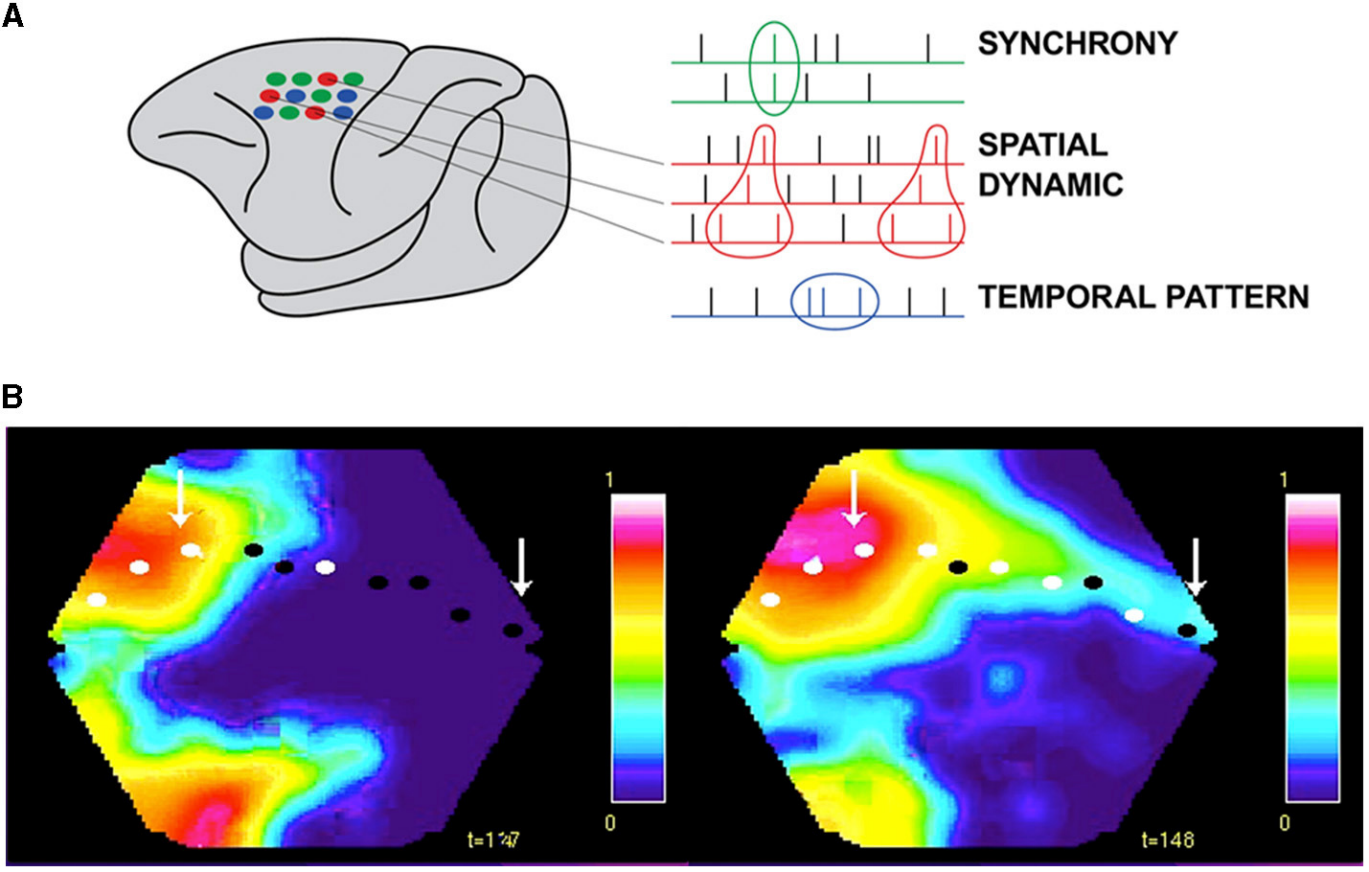
Spatial dynamics of spiking. **(A)** Small groups of individual neurons spike in the same spatial order in single trials from the macaque pre-motor and motor cortex (in contrast to synchrony and temporal patterns, Grün et al., 2022). **(B)** Excitatory sweeps elicited by spiking exciting the dendrites post-synaptically in a spatial order. Left: Excitatory sweep, 122 ms after the appearance of an object moving in the field of view, in areas 19/21 and feedback to areas 17/18. Right: Significant spiking in areas 17/18, mostly in layers 3 and 5, shown by the white spots and excitatory sweep here at 148 ms, ahead of the retinotopical mapping of the moving object (arrow to bright red). The spiking estimating where the object will be mapped in the future (right arrow) and hence where its position in the field of view will be. See the full spatial dynamics in Supplementary Video 2 (isoflurane anesthetized ferret, Harvey et al., 2009).

Working in the premotor and motor cortex of the monkey, Sonja Grün and associates, using rigorous statistics, observed that the same set of neurons in every single trial fired in the same spatial order while the monkey reached out and grasped an object (Grün, 2021; Grün et al., 2022). Subsets of 2–6 neurons elicited from 2 to 6 spikes always in the same spatial order (Figure 8A). These spatial sequences were specific to the components of the reaching task, i.e., related to the cue, delay, preparation, reaching, and grasping (Grün, 2021; Grün et al., 2022). These results show spatial dynamics at the microscopic and single neuron scale. These results cannot be explained as a brain activity using clock time as the independent variable. In contrast, they demonstrate that the timing and order of the spikes depend on the spatial positions of the collaborating single neurons.

Another example violating time as the independent variable in brain processing is when the retinotopic mapping of a moving object co-exists with the mapping of the prediction of its future external position in the visual areas (Figure 8B and Supplementary Video 2).

**Figure 9:**
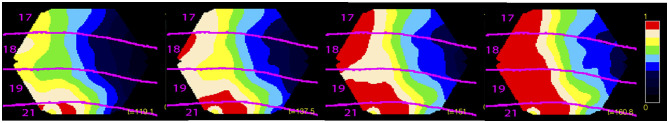
Moving visual object and phase alignment. Object moving downwards from time 0 ms. Phase plot of depolarisation in areas 17, 18, 19, and 21 from six ferrets aligned by their cytoarchitectural borders. Note the leading depolarization in areas 19 and 21 at 119 ms **(left)**. Feedback 137 ms and phase alignment canceling the delays between areas 160.8 ms **(right)** (Harvey et al., 2009).

If an external object moves in the field of view, it is mapped, with different delays, in each retinotopically organized visual area (Supplementary Video 3). So, initially, multiple versions, separated in space and time in the brain, exists of one and the same object. However, higher visual areas convey excitatory feed-back sweeps to lower visual areas aligning the excitation phase between the areas. This cancels their initial separation in brain time and produce unified motion of the object in retinotopical visual areas. This is likely to contribute the experience to perceive only one object moving in the field of view (Figure 9).

**Figure 10:**
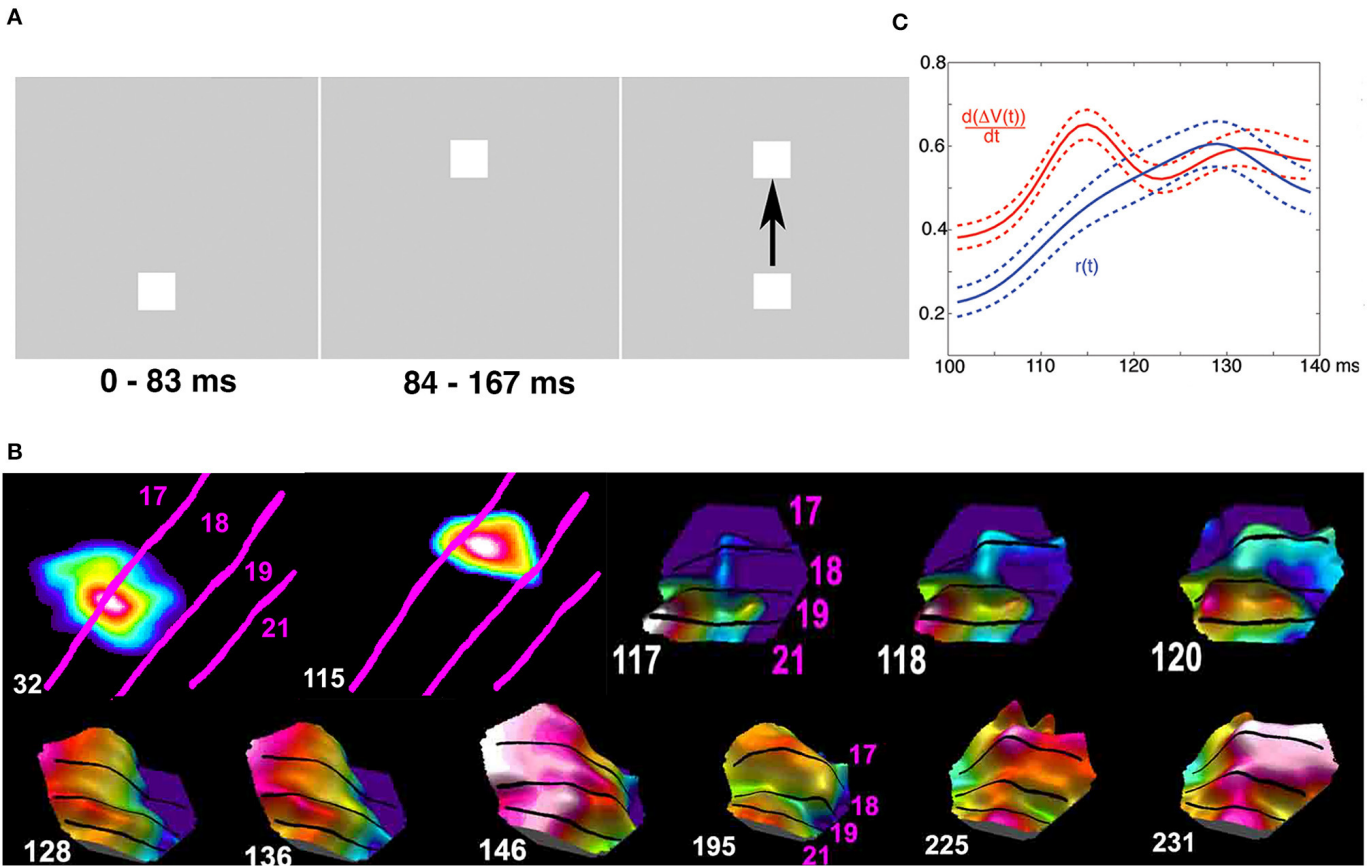
Cortical operations at the mesoscopic scale incompatible with time as an independent variable (apparent motion). Spatial dynamics underlying apparent motion illusion. **(A)** At time 0 ms, the lower object appears. Spiking (not shown) and **(B)** excitation increases map the lower object retinotopically at area 17/18 border at 32 ms. At 83 ms, the upper object appears, and the lower object disappears. The upper object gets mapped at 115 ms retinotopically at a different spatial location along the 17/18 border. At 117 ms, the spiking induces a directed excitation along the 19/21 border (like that for moving objects in Figure 8B) and a feedback excitation to the 17/18 border in between the mapping of the now-extinct lower object and the new upper object. **(C)** At 118 ms, this elicits a directional excitation *d*[Δ*V*(*t*)]/*dt* and spiking *r*(*t*) at the 17/18 border progressing 120 ms to 160 ms in between the former object mapping site and the new (top right). **(B)** The feedbacks then quench the delays between areas, and the cortical excitation proceeds in phase from 146 ms over the 4 areas. The processing in the cortex smoothed space and time and converted two external spatial and temporal distinct objects to one moving object **(A)** (top; modified from Ahmed et al., 2008, licensed under CC BY-NC 2.0).

Brains do not always process stationary objects that are separate in time and space as stationary in time and space (Figure 10A). When first a stationary object appears at one position in the field of view, this is mapped in its retinotopical position in visual areas as explained above. If the first object then disappears and a second stationary object is flashed at another position in the field of view, the second object is mapped (correctly) in its different retinotopical position in the first visual area (Figure 10B). However, the mapping of the second object in higher visual areas elicits spatial-temporal excitatory dynamics smoothing the mapping of the previous object with the present object in brain space (Figure 10B). After this fusion to one object, its dynamics in space and time in the brain is identical to that of a moving object. This elicits the illusion that the first object moved to the new position (Figure 10). Thus, external objects stationary and separate in space and time by brain processing become united to one moving object (apparent motion).

**Figure 11:**
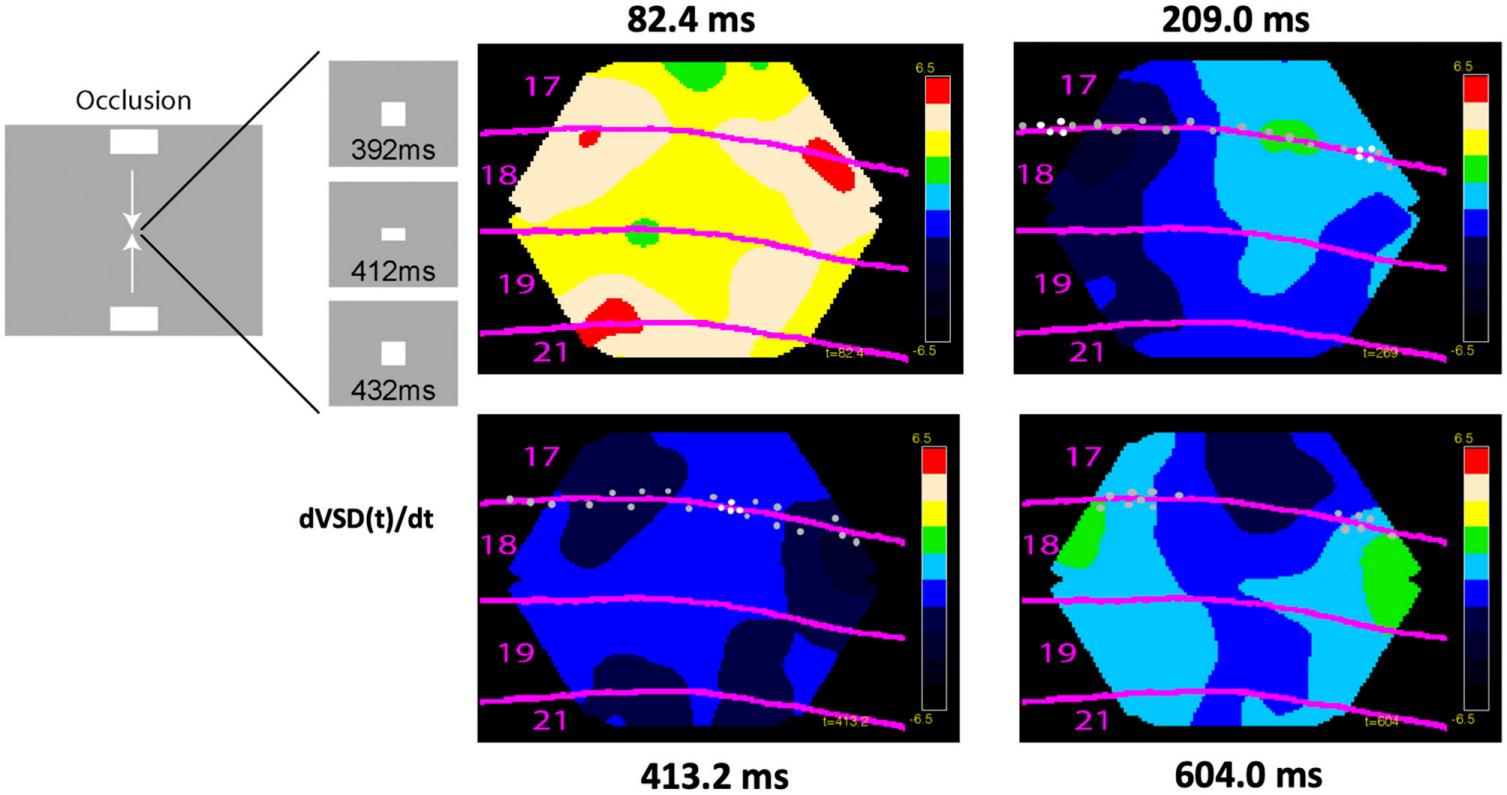
Cortical spiking, excitation, and inhibition at the mesoscopic scale incompatible with time as an independent variable. Excitation, inhibition, and spiking in ferrets exposed to two bars moving to occlude one another in the center of the field of view at 412 ms. Dots show significant spiking and white dots maximal spiking rates, otherwise conventions as in Figure 10. Notice the predictive excitations of the future retinotopic mappings of the objects in areas 17/18 and 19/21 at 82 ms, the maximal spiking at the cortex representing the center of the field of view at 413 ms in an inhibitory regimen of cortical layers 1–3 (data from Harvey and Roland, 2013).

In vision, there is a delay between the appearance of an object until the spiking increases in the first visual area: the retino-cortical delay (Supplementary Video 1). Figure 11 shows how excitatory, inhibitory, and spiking mechanisms in space and time in the brain can quench the perceptual delay by maximizing spiking in the cortex when two oppositely moving objects occlude one-another in the field of view. In the examples shown in Figures 8–11, the ferrets were anesthetized (isoflurane) showing that these brain dynamics were automatic.

These examples demonstrate that all brain activities cannot be explained as evolving with clock time as the independent variable. The examples also illustrate that spiking at the microscopic scale and postsynaptic depolarizations, excitations and inhibitions at the mesoscopic scale evolve with time and space as mutually dependent. The idea of time as an independent variable for brain processes has been criticized from different theoretical points of views (Buzsáki and Tingley, 2018; Gao, 2020; Le Bihan, 2020). For example, interpreting both the meaning of brain responses as measured against the clock in the computer and the meaning of the clock units-might be a fundamental confound in current experimental approach (Buzsáki and Tingley, 2018). Unnecessary assumptions conceptually restrict neuroscience from developing further.

#### 2.6.2. Stationarity

It is often assumed, or claimed, that brain variables end up in some form of stationarity. If this happens, the variable has the same probability distribution over time, i.e., mean, variance, and autocorrelation are invariant over time. If time is not an independent variable for brain processes, the stationarity concept loses its importance in neuroscience. Although stationarities are convenient and simplify mathematics and statistics, are they necessary for understanding brain activities? One may ask then, if the concept of stationarity as defined is invalid for brains, how do brains determine whether external objects are stationary? For vision, Supplementary Video 4 might give a clue. Some 90 ms after the appearance of a stationary object, the spiking, despite continuously changing rates, is confined to the retinotopic map of the object in the primary and secondary visual areas (see also Lamme, 1995). This cannot be explained by statistical and dynamical systems definitions (e.g., fixed point) of stationarity. This is another kind of stationarity, an example of a brain spatial stationarity.

#### 2.6.3. Dynamical systems theory explaining brain activities

A dynamical system is composed of a state space and rules describing the *evolution of the system over time* in this state space. Treating central nervous systems as complex dynamical systems as complex dynamical systems has had some success explaining collective operations of neurons. *In vivo* studies of different spiking networks in the cerebral cortex but also spinal, hypothalamic, and thalamo-cortical networks show the collective spiking dynamics of the network neurons progress as trajectories along low-dimensional, stable manifolds in state space (Churchland et al., 2010; Gallego et al., 2017; Lindén et al., 2022). On the post-synaptic side, field potential, MEG, and EEG studies show state space dynamics like that of strange (chaotic) higher dimensional attractors (Babloyantz and Destexhe, 1986; Stam, 1996; Baria et al., 2017; Willumsen et al., 2022). This dynamic may be identical for all local networks in the human cerebral cortex. However, since the trajectories expand and contract, the dynamic is incompatible with the mathematical definition of attractors (Strogatz, 2018; Willumsen et al., 2022).

Importantly, to be a truly higher dimensional (chaotic) dynamical complex system, the CNS must show sensitivity to initial conditions (Strogatz, 2018). This means that one must determine the initial conditions for a CNS. This requires that for “one point in time,” say within a fraction of a ms, we must know how many variables there are at each point of each neuron (say a point is a membrane surface of 0.1 μm^2^) and which order they have (e.g., higher derivatives of the variables as a result of spatial interaction; Figure 6). We must know exactly where and in which axon or axonal branches action potentials are and know the conduction velocities of each branch (Figure 4). Moreover, as we cannot be sure whether a neuron only has spontaneous ongoing unorganized activity or participates in intrinsic or task-related organized activity, we must know the values of all these variables for all neurons of the CNS within this ms. To define an initial condition in a CNS having ever-ongoing changes of its variables at all spatial scales seems impossible.

Dynamical systems analysis gives the temporal evolution of the collected neurons or local network and neglects spatial interactions. However, one can preserve the locations of the neurons in the data and instead observe the spatial evolution as trajectories in state space (neglecting the temporal evolution) (Roland et al., 2017). Both these approaches thus have limitations. As shown here, dynamical systems theory might not always fit brain activities. The examples in section 2.6.1 show that one can directly observe and measure spatial temporal interactions in the cerebral cortex, instead of analysing temporal and spatial trajectories in abstract state space.

### 2.7. Spatial dynamics, a general hypothesis

The fundamental mechanism of interaction in CNS of most species is spatiotemporal: each neuron sends action potentials through all axon branches to its two–three orders of magnitude more numerous target neurons (Figure 4). This fundamental mechanism creates spatial dynamics in the network of neurons. Postsynaptically, the spatial progress of currents in the dendrites determine plasticity and spike production (Figure 6, section 4.1). Spatial dynamics is a general hypothesis that can be tested experimentally. The hypothesis states that changes in activity variables (section 3) propagate through the network of neurons that makes up a central nervous system. These propagations reveal spatial and temporal interactions underlying CNS activities at different scales (Roland, 2017; Grün et al., 2022). The forces driving the spatio-temporal interactions thus are transmembrane currents, receptor driven, and biochemical. The word dynamics refer to these biophysical and biochemical forces driving the interactions. Thus, spatial dynamics is not related to dynamical systems theories and do not carry any further assumptions about brain activity variables and their interactions.

#### 2.7.1. Spatial dynamics at different scales of observation

**Figure 12:**
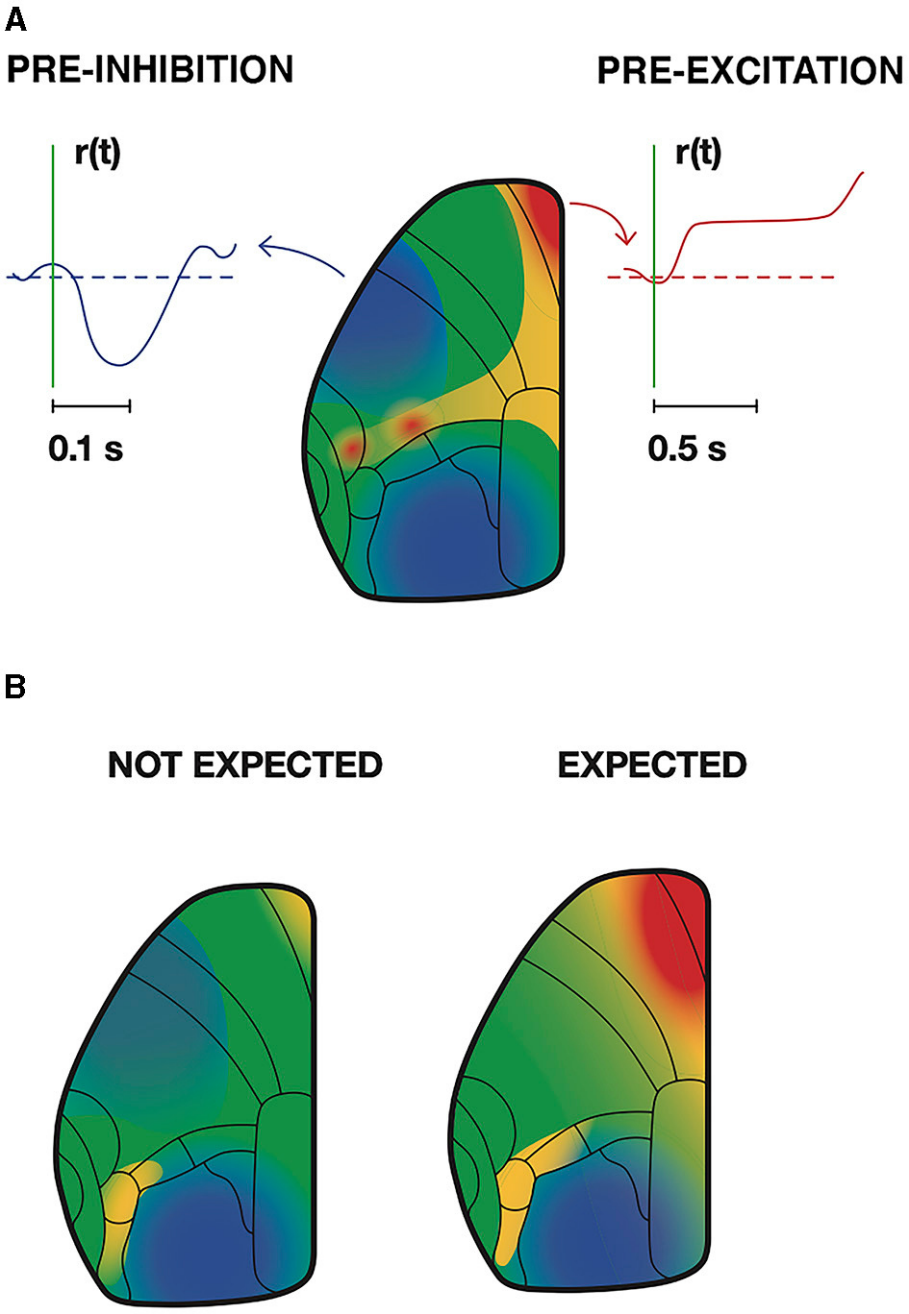
Trained mice inhibit and excite relevant cortical areas prior to stimulation and motor response. **(A)** At the time indicated by the vertical green line, a weak whisker stimulus is given. Intracellular Ca^2+^ and spiking rate, *r*(*t*), decreased in pyramidal neurons in motor and visual areas, but increased in anterior cingulate and pre-motor cortex. However, the mouse must wait 1,000 ms until a beep tells that it can obtain its reward by licking (redrawn from Esmaeili et al., 2021, licensed under CC-BY 4.0). **(B)** Mice continuously watch a moving grating for a sustained change in speed and respond by licking their reward. At periods when such a change was unlikely, this elicited moderate intracellular Ca^2+^ increases in premotor and motor areas in contrast to when the change was expected. Note the intracellular Ca^2+^ decrease in pyramidal neurons' primary visual cortex and increase in visual association areas in advance of the stimulus change (redrawn from Orsolic et al., 2021, licensed under CC-BY 4.0).

Spatial dynamics is not a new idea. Tasaki et al. (1968) used a voltage sensitive dye to follow the course of an action potential. Spatial dynamics has been slowly progressing since then but boosted by recent techniques permitting simultaneous measurements of CNS activity variables in large parts or a whole CNS (see technical obstacles). Figures 8–11 and Supplementary Videos 1–4 are concrete examples of spatial dynamics of spiking and postsynaptic changes in excitation and inhibition leading to visual object perception and the apparent motion illusion. Spatial dynamics of spiking and postsynaptic activities operate in single neurons (Figures 6, 8A) small groups of neurons (Figures 8B, 11), and larger populations of neurons (Figures 8B–12). Spatial derivatives are needed to distinguish different forms of postsynaptic processing at the network scale (Supplementary Videos 1, 5). Spatial dynamics of the activity variables progress though the low-dimensional geometry of a CNS and are therefore wellsuited to reveal mechanisms of neuron interactions at the population (mesoscopic) scale. Its challenge is to find principles to form theories of interactions between multiple neurons.

*Conceptual frontier 10: Use spatial dynamics to find principles of interactions of neurons at all scales of observation*.

#### 2.7.2. Cortical spatial dynamics

Spatial dynamics in the cerebral cortex relate directly to detection, prediction, perception, illusions, retrieval, and consolidation of memories in rodents, carnivores, and primates (Grün et al., 2022) (Figures 8–12). Here it is not the purpose to review spatial dynamics, only to give some concrete examples.

Postsynaptic excitations propagating over dendritic fields may have many shapes and speeds (Supplementary Videos 1–5) (Xu et al., 2007; Mohajerani et al., 2010; Denker et al., 2018; Dickey et al., 2021). Broad postsynaptic net-excitations followed by local net-inhibitions give the impression of a wave propagation though the cortical network. The different forms of (mesoscopic) postsynaptic changes have different roles in brain activities. For example, frequency-modulated sounds elicit a depolarization sweep over the relevant tonalities in the first and secondary auditory areas (Horikawa et al., 1998; Farley and Norena, 2013; Horikawa and Ojima, 2017). Retinal excitatory sweeps induced by a saccade elicit a cortical sweep in V1 matching the direction of motion over the retinal photoreceptors (Slovin et al., 2002).

Waves in different directions appear in mesoscopic recordings of current changes in upper layers of cortex with fast voltage indicators (Prechtl et al., 1997; Senseman, 1999; Roland et al., 2006, 2017; Xu et al., 2007; Mohajerani et al., 2010; Denker et al., 2018; Davis et al., 2020) (Figures 9–11), or in genetically labeled pyramidal excitatory neurons, or as changes in glutamate release (Berger et al., 2007; Song et al., 2018; Abadshi et al., 2020; Zhu et al., 2021). The examples in Figures 8B–11 and Supplementary Videos 1–5 were recordings from isoflurane anesthetized ferrets receiving a visual stimulus. Although the visual stimulus initially drives the cortical neurons after some 28 ms, the cortex does not produce a spatial pattern of the stimulus in each visual area. Rather autonomous cortical spiking and postsynaptic spatial dynamics take over producing lateral spreading excitation, feedback waves and local inhibitions. This dynamics after some 90–120 ms converge to a spatio-temporal “interpretation of the visual surround” in the visual areas. Similarly, the moving visual stimulus initially likely drives the retinotopical depolarization, but autonomus spatial dynamics take over and produce predictive depolarizations and spiking and further spatial dynamics (Supplementary Videos 2, 3).

*Conceptual frontier 11: form hypotheses of how different forms of spatial dynamics distinguish different organized CNS activities*.

#### 2.7.3. Learning dependent spatial dynamics in awake animals

In animals trained to perform a task, intracellular Ca^2+^ can stay increased for longer periods, while in other areas intracellular Ca^2+^ stays decreased for longer periods. These changes are learning and task dependent (Gilad and Helmchen, 2020; Salkoff et al., 2020; Clancy and Mrsic-Flogel, 2021; Liang et al., 2021) (Figure 12). The optical signals reporting these changes stem mainly from the upper, supragranular, layers of cortex. However, there are several examples of discrepancies between spiking and mesoscopic post-synaptic activity, even in supragranular layers. This could be spiking under inhibitory regimes (Orsolic et al., 2021) (see also Figure 11), or no spiking under excitatory post-synaptic regimes, pre-excitation (Roland, 2010) (Figure 12). These discrepancies are in accordance with the earlier mentioned observations that dendrites may be well depolarized without giving rise to action potentials or apical dendrites inhibited while neurons are spiking (section 3.1).

*Conceptual frontier 12: Measuring the spatial dynamics in CNS structures and relate this to measures of excitation and inhibitory spatial postsynaptic dynamics in the same structures and vice versa*.

Generally, spatial dynamics are causal. In naïve animals weak or moderate stimuli may not give rise to a local excitation and spiking in primary sensory areas. If it does, the excitation and spiking do not progress to other areas and structures. This contrasts with well-trained animals. In trained animals, failure of a trial specific spatial dynamics to progress from the primary sensory area to other areas and subcortical structures leads to failure to respond (Gilad and Helmchen, 2020; Salkoff et al., 2020; Esmaeili et al., 2021; Orsolic et al., 2021). Thus, spatial dynamics is likely to propagate such that changes in the activity variables propagate from microscopic scales to engage larger parts of a CNS. However, this does not exclude more restricted local forms of spatial dynamics. Details of how spatial interactions evolve in and between subcortical structures are not known (Figure 5).

*Conceptual frontier 13: reveal the spatial dynamics of subcortical structures at all spatial scales*.

## 3. Technical obstacles

The lack of techniques to follow the course of action potentials through a CNS is often claimed the reason for the lack of progress in systems neuroscience (Bargmann et al., 2014). Given the premise that many parts of a CNS, the brain stem, thalamus, basal ganglia, cerebellum, and the brain itself do seem to participate even in simpler tasks, global access to a CNS seems a must. The axonal diameters of primate cortico-cortical axons range from 0.2 to 4 μm (Liewald et al., 2014). This gives conduction velocities up to 35 mm ms^−1^ (Waxman and Bennett, 1972). In addition, the relevant sampling space in humans range from synapses 0.5 μm^3^ to a human brain hemisphere 700 cm^3^, i.e., 14 orders of magnitude. In comparison, Zebrafish larvae with their translucent CNS and 100,000 neurons with slower axonal conduction of action potentials seem an ideal species for studying spatial CNS dynamics.

The physiologically relevant techniques are electro- physiological, magnetic, and optical. Applications of these techniques in multiple recordings simultaneously from CNS are well described in recent reviews (Engel and Steinmetz, 2019; Cardin et al., 2020; Moreaux et al., 2020; Machado et al., 2022; Urai et al., 2022). So here the focus is on limitations that cannot be solved by combinations of electrophysiological and optical techniques.

Modern multi-electrodes can in principle access all parts of the CNS, yielding spiking from 20,000 to 100,000 neurons simultaneously in animals, and humans with sampling frequencies >20 kHz (Jun et al., 2017; Steinmetz et al., 2019; Paulk et al., 2021). Spike recordings do not reveal the type of neurons involved (excitatory glutamatergic, inhibitory GABAergic, and glycine-ergic sub-types). Moreover, extracellular spike recordings are blind to the dendritic contributions.

Optical recordings can capture dendritic contributions in relevant space-time scales, with voltage-sensitive dyes or genetically encoded voltage sensors (GEVI) with sampling rates op to 2 kHz (Roland et al., 2017; Song et al., 2018; Villette et al., 2019; Moreaux et al., 2020). Intracellular Ca^2+^ changes in single dendrites and single synapses can be detected with recent GCaMP reporters, which are able to capture changes currently at 20 ms scale (50 Hz). This captures slow spatial dynamics, but not the fast (Ferezou et al., 2007; Muller et al., 2016; Grün et al., 2022) (Figures 8–12, Supplementary Videos 1–5). The local interdigitation of dendrites from thousands of neurons (Figure 3) implies that post-synaptic transformation by individual neurons cannot be resolved with one-photon, two-photon, or three-photon optical recordings, because it is difficult to match the active dendritic branches with the right neuron. Labeling all dendritic and axonal terminal branches with voltage sensors gives an overcrowded picture in which this problem takes immense dimensions. In addition, it is a challenge to trace action potentials in thin axonal branches and their origin from neurons in other areas (Figures 3, 4).

*Technical frontier 1: Reveal the spatial dynamics in axonal branches and of synaptic and dendritic processing and connect this to the appropriate neurons*.

Genetically encoded voltage sensors specifically expressed in only one-subclass of neurons make this problem easier to tackle (Abdelfattah et al., 2019; Piatkevich et al., 2019; Villette et al., 2019). In these neurons, one can follow the depolarizations, hyperpolarizations, and progress of action potentials in single trials *in vivo* with 1 kHz sampling rates. It is possible to implant fiber optics and even optical probes providing excitation light and detection of fluorescence along multiple sites on the same probe. However, recordings of dendritic excitation and inhibition dynamics are restricted to the narrow space along the implanted optic probe (Moreaux et al., 2020).

At high resolution, it is possible to selectively examine subclasses of excitatory and inhibitory neurons. However currently, no coherent recordings of a whole insect or mammalian CNS is possible at any spatial scale (Piatkevich et al., 2019; Villette et al., 2019; Cardin et al., 2020; Moreaux et al., 2020; Machado et al., 2022; Urai et al., 2022). Moreover, the genetic incorporation of reporters of membrane current changes, and contributions from neuron subclasses is limited to a few species.

*Technical frontier 2: Including primates is so far out of reach for comprehensive spatial dynamic recordings*.

It is difficult to envisage a noninvasive technique for primates with physiologically relevant sampling frequency. Perhaps, novel MEG-techniques with quantum field sensors and improved depth resolution may develop into tomographic MEG for primate brains (Bezsudnova et al., 2022).

## 4. Experimental obstacles

**Figure 13:**
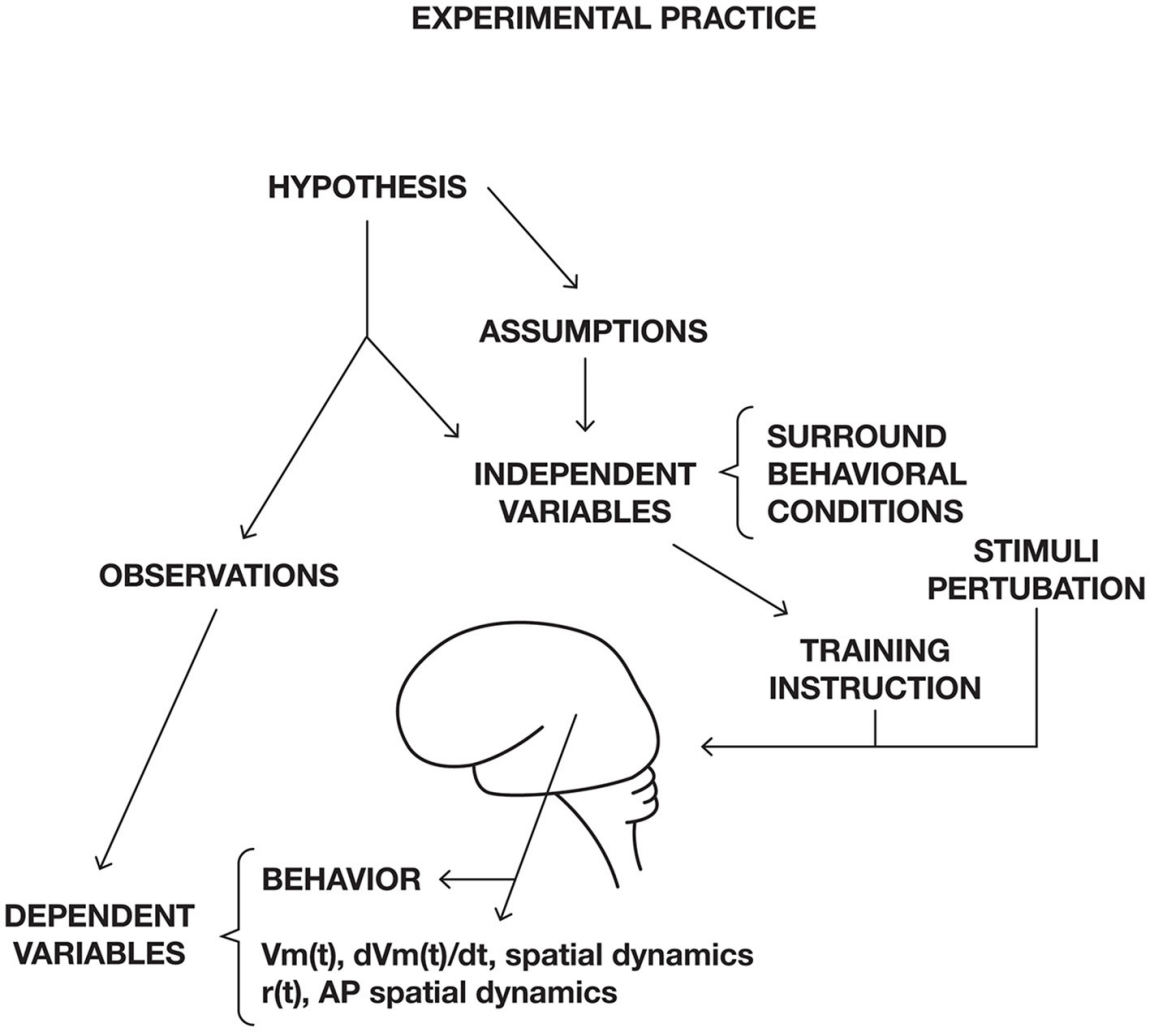
Experimental practice in neuroscience. Dependent variables, for example Membrane potential, *Vm*(*t*), trans-membrane currents *dVm*(*t*)/*dt*, spike rate *r*(*t*), or action potentials, AP, and their spatial dynamics. The experimental trial can start with a cue or a stimulus. During the trial, the experimenter measures dependent variables, for example spike trains and membrane currents or membrane potential changes. The recorded dependent variables are then compared to recordings of the same dependent variables during a baseline or control condition.

Ordinarily, experiments are performed on a CNS to test a hypothesis. The hypothesis is the prediction of the outcome of the experiment. Sometimes, the hypothesis can be quite general. In most experiments, the experimenter determines and manipulates the independent variables. For example, controlling the surround to minimize confounding factors and specifying the behavioral conditions (see conceptual frontier 5; Figure 13).

### 4.1. Baseline and control conditions

Animals must be trained to perform tasks. In the example in Figure 12A, deflection of the whisker at an early stage of training will give no change in intracellular Ca^2+^ in the cortex. After many training trials, intracellular Ca^2+^ and spiking will increase in the primary sensory (barrel) cortex and spread to the secondary sensory cortex and from there to the premotor and motor cortex (Esmaeili et al., 2021; Gallero-Salas et al., 2021). Thus, the prerequisite for the task-induced spatial dynamics is successful learning.

**Figure 14:**
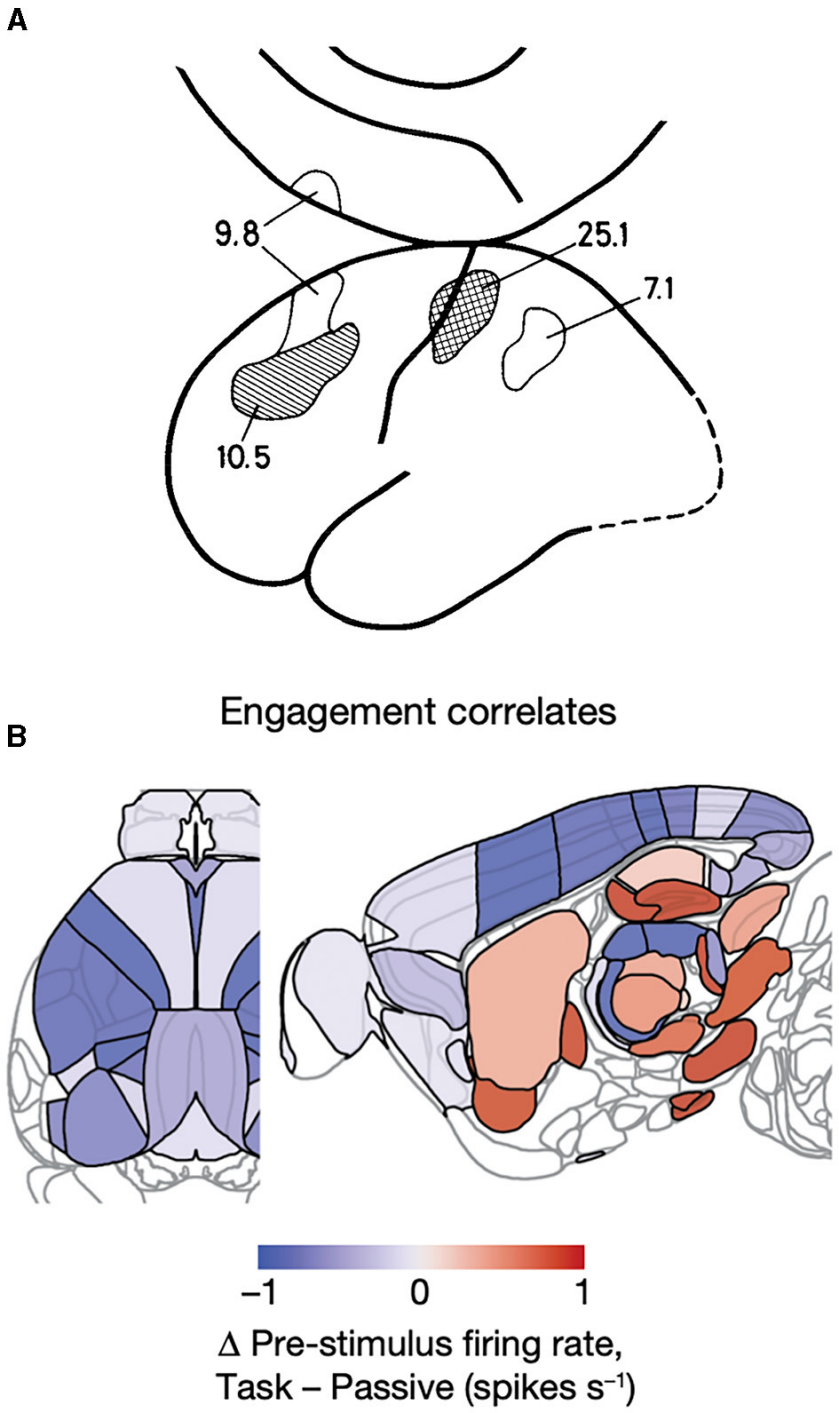
Pre-trial CNS activity. **(A)** Regional cerebral blood flow increases in percent in prefrontal, primary, and parietal somatosensory areas prior to a single trial in which the subject expects a threshold stimulus on the tip of the right index finger compared to physiological defined rest condition (see text) (Roland, 1981). **(B)** Changes in spiking rates prior to experimental trials. Spiking prior to trials (indicating task engagement) of neurons in visual, somatosensory, primary motor, retrosplenial, ACA cortex, and posterior thalamus (LP, PU) correlates negatively with the engagement, but the spiking in nucleus accumbens, globus pallidus ext., CA3 of the hippocampus, dentate gyrus, parafasicular nucleus of thalamus, midbrain reticular formation, and substantia nigra correlates positively with task engagement, if “passive” visual stimulation is taken as baseline condition (from Steinmetz et al., 2019).

When mice have learned a task, spiking increases prior to the experimental trial in CA3 of the hippocampus, dentate gyrus, basal ganglia, zona incerta, substantia nigra, midbrain reticular formation and anticipatory Ca^2+^ increases may appear in specific cortical areas (Steinmetz et al., 2019; Salkoff et al., 2020; Orsolic et al., 2021) (Figure 12). Humans are usually verbally instructed to perform experimental tasks. If they understand the instruction, the regional cerebral blood flow increases in cortical areas engaged in the processing of the sensory stimuli, prior to the experimental trial (Figure 14).

Awake-trained animals and humans are not naïve. In contrast, they are specifically engaged in performing the task *prior* to the experimental trial. Prior to the experimental trial, spatial dynamics evolves in the brain stem, hippocampus, basal ganglia, and cortex. This experimental-related preparatory spatial dynamic probably fine tune the excitability in structures and cortical areas relevant for executing the task (Roland, 1981; Steinmetz et al., 2019; Gilad and Helmchen, 2020; Salkoff et al., 2020; Esmaeili et al., 2021; Orsolic et al., 2021) (Figures 12, 14). These preparatory spatial dynamics may explain how micro-stimulation of singe neurons can control the choice of an animal (Romo et al., 1998; Houweling and Brecht, 2008). Changes in brain variables in most cases are measured relative to a biased pre-stimulus or pre-trial measurements in which the CNS structures to investigate are already active or specifically inhibited.

*Experimental frontier 1: Which reference should measurements from brains have?*

Historically, the field of human brain imaging tried to establish a commonly agreed reference, a defined rest condition. This is a behavioral reference, during which there are no changes in sensory input and no voluntary motor activity, and with physiologically defined reference values of blood pressure, heart rate, galvanic skin response, and EEG pattern (Roland and Larsen, 1976). But the rest condition is also a consequence of an instruction. The assumption that this “rest state” is stationary and valuable as a reference for trials done immediately before or after the rest measurement is most likely false. So, if there are no external or internal stationary references, how should we measure changes in spiking and currents and magnetic signals from brains? Also, how should we interpret the measured changes?

A practical solution is that one could measure where and when changes in membrane currents, magnetic fields, and spiking occur without any internal or prior brain reference. This could also be done during the training of the animals and while humans receive the task instructions.

*Experimental frontier 2: Distinguish different operations in the brain, for example by their spatial dynamics at all scales and in single trials*.

Theoretically, at least, one could get a rough classification of brain activities to start with. Secondly one could relate these data to other changes in brain variables in space and time.

### 4.2. Experimental design, single trials

Single-trial design and analysis is mandatory because brains organize behavior with differences in single trials. The spiking dynamics reflects a single-trial variability (Riehle et al., 2018; Steinmetz et al., 2019; Cowley et al., 2020; Salkoff et al., 2020; Williams and Linderman, 2021). Spatial spiking dynamics is a single-trial dynamics (Grün et al., 2022).

Averaging across neurons, single trials, single areas, or other CNS structures hides the underlying spatial dynamics (Riehle et al., 2018; Davis et al., 2020; Grün et al., 2022). The concepts behind this praxis, behind the experimental design, and behind the interpretation of results are influenced by the separation of time and space. For example, this holds for concepts such as representation, spike pattern, temporal codes, maps, place cell, and synchrony.

The assumptions underlying temporal and spatial averaging, multi-trial statistics, and statistical independence of trials are most likely wrong. So, neuroscientists are forced to design single-trial experiments and analyze single trials statistically (Lee et al., 2010; Rey et al., 2015; Williams and Linderman, 2021).

*Experimental frontier 3: Single-trial statistics*.

Current single-trial statistics make use of a dynamical systems approach. The key to observe differences between single trials is to record simultaneously from many positions/neurons. Often, spike data, membrane, and field potentials are of lower dimensionalities than the number of neurons/positions recorded. So, first one needs to estimate the true dimensionality of the data at hand.

Dimensionality is the number of dimensions one needs to get an exhaustive description of the dynamics of variables in state space. There are several methods by which one can find the dimensionality of time series data. The best method is the Grassberger and Procaccia (1983) method (Camastra, 2003). The end-product is a trajectory of the single-trial behavior in a multidimensional state space of the found true dimensions. Trials with different dynamics evolve in partly different parts of this multidimensional state space (Churchland et al., 2010).

The drawback of this method is that the dimensionality of the state space must be constant for all single trials (Spaak et al., 2017; Willumsen et al., 2022).

## 5. Obstacles in interpretation and explaining CNS operations

In experimental neuroscience, scientists usually measure changes in some dependent brain variables induced by experimental manipulations of independent variables (Figure 13). The measured changes in the observed dependent variables, spiking, membrane potentials, field potentials, magnetic and electrical fields, blood flow, and BOLD signals are interpreted related to external, optogenetic, or direct brain stimulation, particular behaviors, rewards, memory retention, overt behavior, and changes in performance. Careful analyses of the measurements often show that only minor proportions of the variance or information in the data can be explained as related to stimuli, motor behavior, reward behavior, and performance (Urai et al., 2022). This opens several fundamental questions for the interpretation of CNS measurements.

Summarizing the conclusions from the analysis of the barriers hampering progress, the premises for the interpretation of experimental results in systems neuroscience are:

1. Lack of reference or baseline conditions.
2. The continuously changing spiking and changing transmembrane currents everywhere in a CNS implies that one cannot apply a classical cause-effect analysis: if A, t_1_ then B, t_2_.
3. Central nervous systems, in contrast to complex dynamical systems, have no clear initial state definition, neither locally nor globally. This implies that we cannot explain the future states of the system from local or global initial states.
4. Neither can we assume any pre-existing dimensional state space, because dimensionalities change concurrently in many locations in a CNS. This implies that dynamical systems theory may be of limited value.
5. Time is probably not an independent variable for CNS operations. In a CNS, dynamics are space and time dependent, i.e., spatial dynamics. This implies that pooling data from different neurons or locations and temporal and spatial averaging destroy the spatial dynamics. Repeated observations show that spatial dynamics can vary from trial to trial. This in turn implies that conclusions must be drawn from the outcomes of single trials. Moreover, since external clock time does not uniquely relate to the activities of neurons, other types of causality, e.g., Granger causality, are of no help. Assumptions of statistical stationarities of spiking or transmembrane currents are most likely invalid.
6. Referring to external input or motor, behavioral, output has limited explanatory power, because many CNS processes are intrinsic and relatively autonomous.
7. Separating task related activities from spontaneous and intrinsic cognitive activities in a CNS is still difficult.
8. For experiments in humans, introspection is invalid to explain CNS activities, because brains produce experience and motor activity as results of processes lasting from some 120 ms to more than 1,000 ms (Fried, 2022). These spatial dynamics processes, which initially are logically in-accessible, must arrive to some stage of organization before the human subject can report.
9. Current neuroscience is limited to observe spatial dynamics in discrete parts of CNS only.

*Theoretical frontier 1: How can we reliably interpret our results?*
*Theoretical frontier 2: How can we reliably explain our results?*
*Theoretical frontier 3: How can we start to make theories of brains?*

A scientific brain theory would be an experimentally based general explanation on how the elements in brains interact at all scales of observation under all conditions. A theory must serve as a conceptual structure in which gaps of knowledge and inconsistencies can be isolated. It must offer rules and coherent explanations, to some extent encompassing different scales of observation. With the recent technical advances, neuroscience now is free to explore complex brain tasks and conditions in many species. Hopefully, scientists could use their experimental results to find principles which could be part of a brain or CNS theory.

## Author's note

Despite a century of anatomical, physiological, and molecular biological efforts scientists do not know how neurons by their collective interactions produce percepts, thoughts, memories, and behavior. Scientists do not know and have no theories explaining how brains and central nervous systems work. The usual explanations are that scientists lack methods, techniques, and efficient data analysis to obtain this goal. These are no longer the main reasons. The main obstacles for systems neuroscience seem to be conceptual. That is lack of concepts rooted in solid experimental results, unnecessary assumptions, and focus on analogies from other disciplines (information theory, computer science, physics, and psychology). Brains cannot be understood treating time and space as independent variables. Methods are now available for measuring spatial dynamics at microscopic to mesoscopic scales, also in single trials. This paper summarizes the conceptual, theoretical, statistical, and experimental practice obstacles which need to be eliminated to efficiently use and interpret results with these new methods.

## Data availability statement

The original contributions presented in the study are included in the article/Supplementary material, further inquiries can be directed to the corresponding author.

## Author contributions

The author confirms being the sole contributor of this work and has approved it for publication.

## Supplementary material

Supplementary Video 1 Single trial recording of temporal derivative of the voltage signal (showing excitation and inhibition) over visual areas 17, 18, 19, and 21 (see Figures 7, 8). From −180 ms to +20 ms the movie shows spontaneous un-organized spatial fluctuations. From 21 to 200 ms organized spatial excitation and inhibition dynamics in response to a 3° × 3° stationary square at 0 ms, exposed for 133 ms.

Supplementary Video 2 Statistically significant (*p* < 0.01 after Bonferroni correction) depolarization in visual areas of a ferret in response to a bar moving downwards starting in the peripheral field of view. The retina is stationary. Note that the bar then is mapped as moving excitation over the cortex. However, at 104 ms the neurons in area s 19/21 compute an excitation far ahead of the bar mapping. After feedback to areas 17/18 this repeats here. The black holes show the electrode penetration sites along the border between areas 17 and 18 corresponding to the vertical meridian. When the spiking at any layer of the cortex becomes statistically significant (*p* < 0.01) the hole turns white. Note the mapping of the future bar trajectory when the bar representation on the cortex has reached the left white arrow (155 ms). Note also how the object mapping, defined by the hot spot in area 17/18 actually follows the cortical route predicted already at 160 ms. Animal 410 (from Harvey et al., 2009).

Supplementary Video 3 Three-dimensional visualization of derivative of the voltage signal showing excitation (orange to red) and inhibition (dark green to blue) in areas 17, 18, 19, 21 of a ferret to an object moving down from time 0 ms in the field of view. For localization of area borders (see Figure 9) (from the top areas 17, 18, 19, and 21). Note the non-linear spatial dynamics, feedback from areas 21 and 19 to 18 and 17 at 115 ms, predictive excitation 135-195 ms and inhibition chasing the excitations from 500 ms (from Harvey et al., 2009).

Supplementary Video 4 Spiking in layer 4 of areas 17 and 18 of 8 ferrets. Electrode positions are marked with white circles. Color scale shows the proportion of trials giving rise to significant increases (compared to pre-trial baseline). Note that significant spiking gets restricted to the retinotopic mapping after 90 ms (time on top) (from Roland et al., 2017).

Supplementary Video 5 Spatial derivatives in areas 17, 18, 19, 21, to a 3° × 3° stationary square at 0 ms, exposed for 250 ms. Compare with Supplementary Video 1.

## References

[B1] Abadshi J. K. Nazari-Ahangarkolaee M. Gattas S. Bermudez-Contreras E. Luzak A. McNaughton B. L.. (2020). Spatiotemporal patterns of neocortical activity around hippocampal sharp-wave ripples. Elife 9, e51972. 10.7554/eLife.51972 32167467 PMC7096182

[B2] Abdelfattah A. S. Kawashima T. Singh A. Novak O. Liu H. Shuai Y.. (2019). Bright and photostable chemigenetic indicators for extended *in vivo* voltage imaging. Science 365, 699–704. 10.1126/science.aav6416 31371562

[B3] Abeles M. (1991). Corticotronics. Cambridge: Cambridge University Press, 280.

[B4] Ahmed B. Hanazawa A. Undeman C. Eriksson D. Valentiniene S. Roland P. E. (2008). Cortical dynamics subserving visual apparent motion. Cereb. Cortex 18, 2796–2810. 10.1093/cercor/bhn038 18375528 PMC2583157

[B5] Alcami P. El Hady A. (2019). Axonal computations. Front. Cellular Neurosci. 13:413. 10.3389/fncel.2019.00413 PMC6759653 31619963

[B6] Alito H. J. Usrey W. M. (2005). Dynamic prope3rties of thalamic neurons for vision. Prog. Brain Res. 149, 83–90. 10.1016/S0079-6123(05)49007-X 16226578

[B7] Babloyantz A. Destexhe A. (1986). Low-dimensional chaos in an instance of epilepsy. Proc. Natl. Acad. Sci. U. S. A. 83, 35613–33517. 10.1073/pnas.83.10.3513 3085091 PMC323547

[B8] Bargmann C. Newsome W. Anderson D. Brown E. Deisseroth K. Donoghue J. (2014). BRAIN 2025. National Institutes of Health, June 5. Available online at: https://braininitiative.nih.gov/sites/default/files/documents/brain2025_508c_2.pdf

[B9] Baria A. T. Maniscalco B. He B. J. (2017). Initial-state-dependent, robust, transient neural dynamics encode conscious visual perception. PLoS Comput. Biol. 13, e1005806. 10.1371/journal.pcbi.1005806 29176808 PMC5720802

[B10] Barlow H. R. (1961). “Possible principles underlying the transformations of sensory messages,” in Sensory Communication, ed W. A. Rosenblith (Cambridge, MA: MIT Press), 217–234.

[B11] Barth A. L. Poulet J. F. A. (2012). Experimental evidence for sparse firing in the neocortex. Trends Neurosci. 35, 345–355. 10.1016/j.tins.2012.03.008 22579264

[B12] Bassett D. S. Cullen K. E. Eickhoff S. B. Farah M. J. Goda Y. Haggard P.. (2020). Reflections on the past two decades of neuroscience. Nat. Rev. Neurosci. 21, 524–534. 10.1038/s41583-020-0363-6 32879507

[B13] Berger T. Borgdorff A. Crochet S. Neubauer F. B. Lefort S. Fauvet B.. (2007). Combined voltage and calcium epiflourescence imaging *in vitro* and *in vivo* reveals subthreshold and suprathreshold dynamics of mouse barrel cortex. J Neurophysiol. 97, 3751–3762. 10.1152/jn.01178.2006 17360827

[B14] Besle J. Shevon C. A. Mehta A. D. Lakatos P. Goodman R. R. McKhan G. M.. (2011). Tuning of the human neocortex to the temporal dynamics of attended events. J. Neurosci. 31, 3176–3185. 10.1523/JNEUROSCI.4518-10.2011 21368029 PMC3081726

[B15] Bezsudnova Y. Koponen L. M. Barontini G. Jensen O. Kowalczyk A. U. (2022). Optimising the sensing volume of OPM sensors for MEG source reconstruction. Neuroimage 264, 119747. 10.1016/j.neuroimage.2022.119747 36403733 PMC7615061

[B16] Bialeck W. van Steveninck R. R. Rieke F. Warland D. (1997). Spikes, Exploring the Neural Code. Cambridge, MA: MIT Press, 414.

[B17] Buzsaki G. (2019). The Brain Inside Out. Cambridge, MA: MIT Press. 10.1093/oso/9780190905385.001.0001

[B18] Buzsaki G. (2020). The brain-cognitive behavior problem: a retrospective. eNeuro 7, ENEURO.0069-20.2020. 10.1523/ENEURO.0069-20.2020 32769166 PMC7415918

[B19] Buzsáki G. Tingley D. (2018). The hippocampus as a sequence generator. Trends Cogn. Sci. 22, 853–869. 10.1016/j.tics.2018.07.006 30266146 PMC6166479

[B20] Camastra F. (2003). Data dimensionality estimation methods: a survey. Pattern Recogn. 36, 2945–2954. 10.1016/S0031-3203(03)00176-6

[B21] Cardin J. A. Crair M. C. Hogley M. J. (2020). Mesoscopic imaging: shining a wide light on large-scale neural dynamics. Neuron 108, 33–43. 10.1016/j.neuron.2020.09.031 33058764 PMC7577373

[B22] Churchland M. M. Yu B. M. Cunningham J. P. Sugrue L. P. Cohen M. R. Corrado G. S.. (2010). Stimulus onset quenches neural variability: a widespread cortical phenomenon. Nat. Neurosci. 13, 369–378. 10.1038/nn.2501 20173745 PMC2828350

[B23] Clancy K. R. Mrsic-Flogel T. D. (2021). The sensory representation of causally controlled objects. Neuron 109, 677–689. 10.1016/j.neuron.2020.12.001 33357383 PMC7889580

[B24] Cowley B. R. Snyder A. C. Acar K. Williamson R. C. Yu B. M. Smith M. A. (2020). Slow drift of neural activity as a signature of impulsivity in macaque visual and pre-fronal cortex. Neuron 108, 551–567. 10.1016/j.neuron.2020.07.021 32810433 PMC7822647

[B25] d'Aquin S. Szonyi A. Mahn M. Krabbe S. Gründemann J. Lüthi A. (2022). Compartmentalized dendritic plasticity during associative learning. Scence 376, 266. 10.1126/science.abf7052 35420958

[B26] Davis Z. W. Muller L. Martinez-Trujillo J. Sejnowski T. J. Reynolds J. H. (2020). Spontaneous travelling cortical waves gate perception in behaving primates. Nature 587, 432–436. 10.1038/s41586-020-2802-y 33029013 PMC7677221

[B27] Dayan P. Abbott L. F. (2001). Theoretical Neuroscience. Cambridge, MA: MIT Press.

[B28] Denker M. Zehl L. Kilavik B. E. Diesmann M. Brochier T. Riehle A.. (2018). LFP beta amplitude is linked to mesoscopic spatio-temporal phase patterns. Sci. Rep. 8, 5200. 10.1038/s41598-018-22990-7 29581430 PMC5980111

[B29] Dickey C. W. Sargsyan A. Madsen J. R. Eskandar E. N. Cash S. S. Halgren E. (2021). Traveling spindles create necessary conditions for spike -timing-dependent plasticity in humans. Nat. Commun. 12, 1027. 10.1038/s41467-021-21298-x 33589639 PMC7884835

[B30] Eliasmith C. Stewart T. C. Choo X. Bekolay T. DeWolf T. Tang Y.. (2012). A large-scale model of the brain. Science 338, 1202–1205. 10.1126/science.1225266 23197532

[B31] Engel T. A. Steinmetz N. A. (2019). New perspectives on dimensionality and variability from large-scale cortical dynamics. Curr. Opin. Neurobiol. 58, 181–190. 10.1016/j.conb.2019.09.003 31585331 PMC6859189

[B32] Eriksson D. Valentiniene S. Papaioannou S. (2010). Relating information, encoding and adaptation: decoding the population firing rate in visual areas 17/18 in response to a stimulus transition. PLoS ONE 5, e10327. 10.1371/journal.pone.0010327 20436907 PMC2860500

[B33] Esmaeili V. Tamura K. Muscinelli S. P. Modirshanechi A. Boscaglia M. Lee A. B.. (2021). Rapid suppression and sustained activation of distinct cortical regions for a delayed sensory-triggered motor response. Neuron 109, 2183–2201. 10.1016/j.neuron.2021.05.005 34077741 PMC8285666

[B34] Esteves I. M. Chang H. R. Neumann A. R. Sun J. J. Mohajerani M. H. McNaughton B. L. (2021). Spatial information encoding across multiple neocortical regions depends on an intact hippocampus. J. Neurosci. 41, 307–319. 10.1523/JNEUROSCI.1788-20.2020 33203745 PMC7810652

[B35] Farley B. J. Norena A. J. (2013). Spatiotemporal cordiunation of slow-wave ongoing activity across auditory areas. J. Neurosci. 33, 3299–3310. 10.1523/JNEUROSCI.5079-12.2013 23426658 PMC6619509

[B36] Ferezou I. Haiss F. Gentet L. J. Aronoff R. Weber B. Petersen C. C. H.. (2007). Spatiotemporal dynamics of cortical sensorimotor integration in behaving mice. Neuron 56, 907–923. 10.1016/j.neuron.2007.10.007 18054865

[B37] Foster N. N. Barry J. Korobkova L. Garcia L. Gao L. Becerra M.. (2021). The mouse cortico-basal ganglia-thalamic network. Nature 598, 188–194. 10.1038/s41586-021-03993-3 34616074 PMC8494639

[B38] Fried I. (2022). Neurons as will and representation. Nat. Rev. Neurosci. 23, 104–114. 10.1038/s41583-021-00543-8 34931068 PMC9359715

[B39] Fried I. Mukamel R. Kreiman G. (2011). Internally generated preactivation of single neurons in human medial frontal cortex predicts volition. Neuron 69, 548–562. 10.1016/j.neuron.2010.11.045 21315264 PMC3052770

[B40] Gallego J. A. Perich M. G. Miller L. E. Solla S. A. (2017). Neural manifolds for the control of movement. Neuron 94, 978–984. 10.1016/j.neuron.2017.05.025 28595054 PMC6122849

[B41] Gallero-Salas Y. Han S. Sych Y. Voigt F. F. Laurenczy B. Gilad A.. (2021). Sensory and behavioral components of neorcortical signal flow in discrimination with short-term memory. Neuron 109, 135–148. 10.1016/j.neuron.2020.10.017 33159842

[B42] Gao R. (2020). Neuronal timescales are functionally dynamic and shaped by cortical microarchitecture. Elife 9, e61277. 10.7554/eLife.61277.sa2 33226336 PMC7755395

[B43] Gilad A. Helmchen F. (2020). Spatiotemporal refinement of signal flow through assocoiation cortex during learning. Nat. Commun. 11, 1744. 10.1038/s41467-020-15534-z 32269226 PMC7142160

[B44] Goetz L. Roth A. Häusser M. (2021). Active dendrites enable strong but sparse input to determine orientation selectivity. Proc. Natl. Acad. Sci. U. S. A. 118, e2017339118. 10.1073/pnas.2017339118 34301882 PMC8325157

[B45] Gomez-Ramirez M. Kelly S. P. Molholm S. Schatpour P. Schwartz T. H. Foxe J. J.. (2011). Oscillatory sensory selection mechanisms during intersensory attention to rhythmic auditory and visual inputs: a human electrocorticographic investigation. J. Neurosci. 31, 18556–18567. 10.1523/JNEUROSCI.2164-11.2011 22171054 PMC3298747

[B46] Grassberger P. Procaccia I. (1983). Characterization of strange attractors. Phys. Rev. Lett. 50, 346–349. 10.1103/PhysRevLett.50.346

[B47] Gray C. M. Singer W. (1989). Stimulus-specific neuronal oscillations in orientation columns of cat visual cortex. Proc. Natl. Acad. Sci. U. S. A. 86, 1698–1702. 10.1073/pnas.86.5.1698 2922407 PMC286768

[B48] Grün S. (2021). Significant spatio-temporal spike patterns in macaque monkey motor cortex. J. Comput. Neurosci. 49(Suppl 1), S4–S5. 10.1007/s10827-021-00801-9

[B49] Grün S. Li J. McNaughton B. Petersen C. McCormick D. Robson D.. (2022). Emerging principles of spacetime in brains: meeting report on spatial neurodynamics. Neuron 110, 1894–1898. 10.1016/j.neuron.2022.05.018 35709696

[B50] Harris K. M. Hubbard D. D. Kuwajima M. Abraham W. C. Bourne J. N. Bowden J. B.. (2022). Dendritic spine density scales with microtubule number in rat hippocampal dendrites. Neuroscience 489, 84–97. 10.1016/j.neuroscience.2022.02.021 35218884 PMC9038701

[B51] Harvey M. A. Roland P. E. (2013). Laminar firing and membrane dynamics in four visual areas exposed to two objects moving to occlusion. Front. Syst. Neurosci. 7, 23. 10.3389/fnsys.2013.00023 23805082 PMC3691547

[B52] Harvey M. A. Valentiniene S. Roland P. E. (2009). Cortical membrane potential dynamics and laminar firing during object motion. Front. Syst. Neurosci. 3, 7. 10.3389/neuro.06.007.2009 19753323 PMC2742661

[B53] Heller J. Hertz J. A. Kjær T. W. Richmond B. J. (1995). Information flow and temporal coding in primate pattern vision. J. Comp. Neurosci. 2, 175–193. 10.1007/BF00961433 8521286

[B54] Herath P. Klingberg T. Young J. Amunts K. Roland P. (2001). Neural correlates of dual task interference can be dissociated from those of divided attention: an fMRI study. Cereb Cortex. 11, 796–805. 10.1093/cercor/11.9.796 11532885

[B55] Horikawa J. Nasu M. Taniguchi I. (1998). Optical recording of responses to grequency-modulated sounds in the auditory cortex. Neuroreport 9, 799–802. 10.1097/00001756-199803300-00006 9579668

[B56] Horikawa J. Ojima H. (2017). Cortical activation patterns evoked by temporally asymmetric sounds and their modulation by learning. eNeuro 4, ENEURO.0241-16.2017. 10.1523/ENEURO.0241-16.2017 28451640 PMC5399754

[B57] Houweling A. R. Brecht M. (2008). Behavioral report of single neuron stimulation in somatosensory cortex. Nature 451, 65–68. 10.1038/nature06447 18094684

[B58] Izhikevich E. M. Edelmann G. M. (2008). Large-scale model of mammalian thalamocortical systems. Proc. Natl. Acad. Sci. U. S. A. 105, 3593–3598. 10.1073/pnas.0712231105 18292226 PMC2265160

[B59] Jones I. S. Kording K. P. (2022). Do biological constraints impair dendritic computation? Neuroscience 489, 282–274. 10.1016/j.neuroscience.2021.07.036 34364955 PMC8835230

[B60] Jun J. Steinmetz N. A. Harris K. D. Koch C. O'Keefe J. Harris T. D.. (2017). Fully integrated silicon probes for high-density recording of neural activity. Nature 551, 232–236. 10.1038/nature24636 29120427 PMC5955206

[B61] Jung Y. Kennedy A. Chiu H. Mohammed F. Claridge-Chang A. Anderson D. J. (2020). Neurons that function within an integrator to promote a persistent behavioral state in *Drosophila*. Neuron 105, 322–333. 10.1016/j.neuron.2019.10.028 31810837 PMC6981076

[B62] Karimi A. Odenthal J. Drawitsch F. Boergens K. M. Helmstaedter M. (2020). Cell-type specific innervation of cortical pyramidal cells at their apical dendrites. Elife 9, e46876. 10.7554/eLife.46876.sa2 32108571 PMC7297530

[B63] Keyser C. Logothetis N. K. Panzieri S. (2010). Millisecond encoding precision of auditory cortex neurons. Proc. Natl. Acad. Sci. U. S. A. 107, 16976–16981. 10.1073/pnas.1012656107 20837521 PMC2947890

[B64] Kinomura S. Larsson J. Gulyás B. Roland P. E. (1996). Attention activates the midbrain reticular formation and thalamic intralaminar nuclei in man. Science 271, 512–515. 10.1126/science.271.5248.512 8560267

[B65] Kumar A. Schrader S. Aertsen A. (2008). The high conductance state of cortical networks. Neural Comp. 20, 1–43. 10.1162/neco.2008.20.1.1 18044999

[B66] Lamme V. A. F. (1995). The neurophysiology of figure-ground segregation in primary visual cortex. J. Neurosci. 15, 1605–1615. 10.1523/JNEUROSCI.15-02-01605.1995 7869121 PMC6577835

[B67] Larkum M. E. Wu J. Duverdin S. A. Gidon A. (2022). The guide to dendritic spikes of the mammalian cortex *in vitro* and *in vivo*. Neuroscience 489, 15–33. 10.1016/j.neuroscience.2022.02.009 35182699

[B68] Le Bihan D. (2020). On time and space in the brain: a relativistic Pseudo-diffusion framework. Brain Multiphys. 1, 100016. 10.1016/j.brain.2020.100016

[B69] Lee J. Kim H. R. Lee C. (2010). Trial-to-trial variability of spike response of V1 and saccadic response time. *J*. Neurophysiol. 104, 2556–2572. 10.1152/jn.01040.2009 20810695

[B70] Li N. Mrsic-Flogel T. D. (2020). Cortic-cerebellar interactions during goal-directed behavior. Curr. Opin. Neurobiol. 65, 27–37. 10.1016/j.conb.2020.08.010 32979846 PMC7770085

[B71] Liang Y. Song C. Liu M. Gong P. Zhou C. Knöpfel T. (2021). Cortex-wide dynamics of intrinsic electrical activities: propagating waves and their interactions. J. Neurosci. 41, 3665–3678. 10.1523/JNEUROSCI.0623-20.2021 33727333 PMC8055070

[B72] Liewald D. Miller R. Logothetis N. Wagner H.-J. Schüz A. (2014). Distribution of axon diameters in cortical white matter. An electron-microscopic study on three human brains and a macaque. Biol. Cybern. 108, 541–537. 10.1007/s00422-014-0626-2 25142940 PMC4228120

[B73] Lindén H. Petersen P. C. Vestergaard M. Berg R. W. (2022). Movement is governed by rotational population dynamics in spinal motor networks. Nature 610, 526–531. 10.1038/s41586-022-05293-w 36224394

[B74] Machado T. A. Kauvar I. V. Deisserroth K. (2022). Multiregion neuronal activity: the forrest and the trees. Nat. Rev. Neurosci. 23, 683–704. 10.1038/s41583-022-00634-0 36192596 PMC10327445

[B75] Markram H. Muller E. Ramaswamy S. Reimann M. W. Abdellah M. Sanchez C. A.. (2016). Reconstruction and simulation of neocortical microcircuity. Cell 163, 456–492. 10.1016/j.cell.2015.09.029 26451489

[B76] Marques J. C. Li M. Schaak D. Robson D. N. Li J. M. (2020). Internal state dynamics shape brainwide activity and foraging behaviour. Nature 577, 239–243. 10.1038/s41586-019-1858-z 31853063

[B77] McCormick D. A. Nestvogel D. B. He B. J. (2020). Neuromodulation of brain state and behavior. Ann. Rev. Neurosci. 43, 391–415. 10.1146/annurev-neuro-100219-105424 32250724 PMC12237593

[B78] Mel B. (1993). Synaptic integration in an excitable dendritic tree. J. Neurophysiol. 70, 1086–1101. 10.1152/jn.1993.70.3.1086 8229160

[B79] Merchant H. Averbeck B. R. (2017). The computational and neural basis of rhythmic timing in medial premotor cortex. J. Neurosci. 37, 4552–4564. 10.1523/JNEUROSCI.0367-17.2017 28336572 PMC6596663

[B80] Millner P. M. (1999). The Autonomous Brain: A Neural Theory of Attention and Learning. Mawah, NJ: Lawrence Earlbaum Ass. Publishers. 10.4324/9781410602985

[B81] Mohajerani M. H. McVea D. A. Fingas M. Murphy T. H. (2010). Mirroed bilateral slow-wave cortical activity within local circuits revealed by fast bihemispheric voltage-sensitive dye imaging in anesthetized and awake mice. J. Neurosci. 30, 3745–3751. 10.1523/JNEUROSCI.6437-09.2010 20220008 PMC6632233

[B82] Moore J. J. Robert V. Rashid S. K. Basu J. (2022). Assessing local and branch-specific activity in dendrites. Neuroscience 489, 143–164. 10.1016/j.neuroscience.2021.10.022 34756987 PMC9125998

[B83] Moreaux L. C. Yatsenko D. Sacher W. D. Choi J. Lee C. Kubat N. J.. (2020). Integrated neurophotonics: toward dense volumetric interrogation of brain circuit activity-at depth and in real time. Neuron 108, 66–92. 10.1016/j.neuron.2020.09.043 33058767 PMC8061790

[B84] Muller L. Plantoni G. Koller D. Cash S. S. Halgren E. Sejnowski T. J. (2016). Rotating waves during human sleep organize global patterns of activity that repeat precisely though the night. Elife 5, e17267. 10.7554/eLife.17267 27855061 PMC5114016

[B85] Olshausen B. A. Field D. J. (2006). “What is the other 85 percent of V1 doing?” in 23 Problems in Systems Neuroscience, eds J. L. van Hemmen, and T. J. Sejnowski (New York, NY: Oxford University Press), 182–−221. 10.1093/acprof:oso/9780195148220.003.0010

[B86] Orsolic I. Rio M. Mrsic-Flogel T. D. Znamensky P. (2021). Mesoscale dynamics reflect the interaction and temporal expectation during perceptual decision-making. Neuron 109, 1–15. 10.1016/j.neuron.2021.03.031 33861941 PMC8186564

[B87] Otor Y. Achvat S. Cermak N. Benisty H. Abboud M. Barak O.. (2022). Dynamic compartmental computations in tuft dendrites of layer 5 neurons during motor behavior. Science 376, 267–275. 10.1126/science.abn1421 35420959

[B88] Paulk A. C. Kfir Y. Khanna A. R. Mustroph M. L. Trautmann E. M. Soper D. J.. (2021). Large-scale neural recordings with single neuron resolution using Neuropixels probes in huiman cortex. Nat. Neurosci. 25, 252–263. 10.1038/s41593-021-00997-0 35102333

[B89] Peters A. J. Fabre J. M. J. Steinmetz N. A. Harris K. D. Carandini M. (2021). Striatal activity topographically reflects cortical activity. Nature 591, 420–425. 10.1038/s41586-020-03166-8 33473213 PMC7612253

[B90] Pfurtscheller G. Solis-Escalante T. Barry R. J. Klobassa D. S. Neuper C. Müller-Putz G. R. (2013). Brisk heart rate and EEG changes during execution and withholding of cue-paced foot motor imagery. Front. Hum. Neurosci. 7, 379. 10.3389/fnhum.2013.00379 23908614 PMC3726939

[B91] Piatkevich K. D. Bensussen S. Tseng H.-A. Shroff S. N. Lopez-Huerta V. G. Park D.. (2019). Population imaging of neural activity in awake behving mice. Nature 574, 413–417. 10.1038/s41586-019-1641-1 31597963 PMC6858559

[B92] Prechtl J. C. Cohen L. B. Pesaran B. Mitra P. P. Kleinfeld D. (1997). Visual stimuli induce waves of electrical activity in turtle cortex. Proc. Natl. Acad. Sci. USA 94, 7621–7626. 10.1073/pnas.94.14.7621 9207142 PMC23872

[B93] Rao R. P. N. Ballard D. H. (1999). Predictive coding in the visual cortex. a functional interpretation of some extra-classical receptive-field effects. Nat. Neurosci. 2, 79–87. 10.1038/4580 10195184

[B94] Rey H. G. Ahmadi M. Quiroga R. Q. (2015). Single trial analysis of field potentials in perception, learning and memory. Curr. Opin. Neurobiol. 31, 148–155. 10.1016/j.conb.2014.10.009 25460071

[B95] Richmond B. J. Optican L. M. (1990). Temporal encoding of two-dimensional patterns by single units in primate primary visual cortex. II. Information transmission. J. Neurophysiol. 64, 370–380. 10.1152/jn.1990.64.2.370 2213123

[B96] Riehle A. Brochier T. Nawrot M. Grün S. (2018). Behavioral context determines network state and variability dynamics in monkey motor cortex. Front. Neural Circuits 12, 52. 10.3389/fncir.2018.00052 30050415 PMC6052126

[B97] Rockland K. S. (2010). Five points on columns. Front. Neuroanat. 4, 22. 10.3389/fnana.2010.00022 20589097 PMC2893004

[B98] Rockland K. S. (2021). A closer look at cortico-thalamic “loops”. Front. Neural Circuits 15, 632668. 10.3389/fncir.2021.632668 33603649 PMC7884447

[B99] Roland P. E. (1981). Somatotopical tuning on the postcentral gyrus during focal attention in man. A regional cerebral blood flow study. J. Neurophysiol. 46, 744–754. 10.1152/jn.1981.46.4.744 7288462

[B100] Roland P. E. (2010). Six principles of visual cortical dynamics. Front. Syst. Neurosci. 4, 28. 10.3389/fnsys.2010.00028 20661451 PMC2906257

[B101] Roland P. E. (2017). Space-time dynamics of membrane currents evolve to shape excitation, spiking, and inhibition in the cortex at small and large scales. Neuron 94, 934–942. 10.1016/j.neuron.2017.04.038 28595049

[B102] Roland P. E. Bonde L. H. Forsberg L. Harvey M. (2017). Breaking the excitation-inhibition balance makes the cortical network's space-time dynamics distinguish simple visual scenes. Front. Syst. Neurosci. 11, 14. 10.3389/fnsys.2017.00014 28377701 PMC5360108

[B103] Roland P. E. Hanazawa A. Undeman C. Eriksson D. Tompa T. Nakamura H.. (2006). Cortical feedback depolarization waves: a mechanism of top-down influence on early visual areas. Proc. Natl. Acad. Sci. USA 103, 12586–12591. 10.1073/pnas.0604925103 16891418 PMC1531644

[B104] Roland P. E. Larsen B. (1976). Focal increase of cerebral blood flow during stereognostic testing in man. Arch. Neurol. 33, 551–558. 10.1001/archneur.1976.00500080029005 942312

[B105] Romo R. Hernández A. Zainos A. Salinas E. (1998). Somatosensory discrimination based on cortical microstimulation. Nature 392, 387–390. 10.1038/32891 9537321

[B106] Rovelli C. (2018). The Order of Time. London: Penguin Random House.

[B107] Rudolph M. Pospischil M. Timofeev I. Destexhe A. (2007). Inhibition determines membrane potential dynamics and controls action potential generation in awake and sleeping cat cortex. J Neurosci. 27, 5280–5290. 10.1523/JNEUROSCI.4652-06.2007 17507551 PMC6672346

[B108] Salkoff D. A. Zagha E. McCarthy E. McCormick D. (2020). Movement and performance explain widespread cortical activity in a visual detection task. Cereb. Cortex 30, 421–437. 10.1093/cercor/bhz206 31711133 PMC7029483

[B109] Scheffer L. K. Xu C. S. Januszewski M. Lu Z. Takemura S.-Y. Hayworth K. J.. (2020). A connectome and analysis of the adult *Drosophila* central brain. Elife 9, e57443. 10.7554/eLife.57443.sa2 32880371 PMC7546738

[B110] Schmidt M. Bakker R. Shen K. Bezgin G. Diesmann M. van Albada S. J. (2018). A multi-scale layer-resolved spiking network model of resting-state dynamics in macaque visual cortical areas. PLoS Comput. Biuol. 14, e1006359. 10.1371/journal.pcbi.1006359 30335761 PMC6193609

[B111] Senseman D. M. (1999). Spatiotemporal structure of depolarization spread in cortical pyramidal cell populations evoked by diffuse retinal light flashes. Vis. Neurosci. 16, 65–79. 10.1017/S0952523899161030 10022479

[B112] Shepherd G. M. Grillner S. (2018). Handbook of Brain Microcircuits. New York, NY: Oxford University Press, 599. 10.1093/med/9780190636111.001.0001

[B113] Shepherd G. M. Yamawaki N. (2021). Untangling the cortico-thalamo-cortical loop: cellular pieces of a knotty circuit puzzle. Nat. Rev. Neurosci. 22, 389–406. 10.1038/s41583-021-00459-3 33958775 PMC9006917

[B114] Shoham S. O'Connor D. H. Segev R. (2006). How silent is the brain: is there a “dark matter” problem in neuroscience? J. Comp. Physiol. A 192, 777–784. 10.1007/s00359-006-0117-6 16550391

[B115] Siegle J. H. Jia X. Durand S. Gale S. Bennett C. Graddis N.. (2021). Survey of spiking in the mouse visual system reveals functional hierarchy. Nature 592, 86–92. 10.1038/s41586-020-03171-x 33473216 PMC10399640

[B116] Singer W. Sejnowski T. J. Rakic P. (eds) (2019). The Neocortex. Cambridge, MA: MIT Press. 10.7551/mitpress/12593.001.0001

[B117] Slovin H. Arieli A. Hildesheim R. Grinvald A. (2002). Long-term voltage-sensitive dye imaging reveals cortical dynamics in behaving monkeys. J. Neurophysiol. 88, 3421–3438. 10.1152/jn.00194.2002 12466458

[B118] Song C. Piscopo D. M. Niell C. M. Knöpfel T. (2018). Cortical signatures of wakeful somatosensory processing. Sci. Rep. 8, 11977. 10.1038/s41598-018-30422-9 30097603 PMC6086870

[B119] Spaak E. de Lange F. P. Jensen O. (2014). Local entrainment of alpha oscillations by visual stimuli causes cyclic modulation of perception. J. Neurosci. 34, 3636–3544. 10.1523/JNEUROSCI.4385-13.2014 24599454 PMC6608988

[B120] Spaak E. Watanabe K. Funahashi S. Stokes M. G. (2017). Stable and dynamic coding for working memory in primate prefrontal cortex. J. Neurosci. 37, 6502–6516. 10.1523/JNEUROSCI.3364-16.2017 28559375 PMC5511881

[B121] Stam C. J. (1996). Non-linear dynamic analysis of EEG and MEG: review of an emerging field. Clin. Neurophysiol. 116, 2266–2301. 10.1016/j.clinph.2005.06.011 16115797

[B122] Steinmetz N. A. Zatka-Haas P. Carandini M. Harris K. D. (2019). Distributed coding of choice, action and engagement across the mouse brain. Nature 576, 266–273. 10.1038/s41586-019-1787-x 31776518 PMC6913580

[B123] Stringer C. Pachitariu M. Steinmetz N. Carandini M. Harris K. D. (2019). High-dimensional geometry of population responses in visual cortex. Nature 571, 361–365. 10.1038/s41586-019-1346-5 31243367 PMC6642054

[B124] Strogatz S. H. (2018). Nonlinear Dynamics and Chaos, 2 ed. New York, NY: CRC Press. 10.1201/9780429492563

[B125] Stuyt G. Godenzini L. Palmer L. M. (2022). Local and global dynamics of dendritic activity in the pyramidal neuron. Neuroscience 489, 176–184. 10.1016/j.neuroscience.2021.07.008 34280492

[B126] Tasaki I. Watanabe A. Sandlin R. Carnay L. (1968). Changes in in fluoresence, turbidity, birefringence, associated with nerve excitation. Proc. Natl. Acad. Sci. U.S.A. 61, 883–888. 10.1073/pnas.61.3.883 4301149 PMC305410

[B127] Urai A. E. Doiron B. Leifer A. M. Churchland A. K. (2022). Large-scale neural recordings call for new insights to link brain and behavior. Nat. Neurosci. 25, 11–19. 10.1038/s41593-021-00980-9 34980926

[B128] Villette V. Chavarha M. Dimov I. K. Bradley J. Pradhan L. Mathieu B.. (2019). Ultrafast two-photon imaging of a high-gain voltage indicator in awake behaving mice. Cell 179, 1590–1608. 10.1016/j.cell.2019.11.004 31835034 PMC6941988

[B129] Wagner M. J. Kim T. H. Kadmon J. Nguyen N. D. Ganguli S. Schnitzer M. J.. (2019). Shared cortex-cerebellum dynamics in the execution and learning of a motor task. Cell 177, 669–682. 10.1016/j.cell.2019.02.019 30929904 PMC6500577

[B130] Waxman S. G. Bennett N. (1972). Relative conduction velocities of small myelinated and non-myelinated fibres in the central nervous system. Nat. New Biol. 238, 217–219. 10.1038/newbio238217a0 4506206

[B131] Williams A. H. Linderman S. W. (2021). Statistical neuroscience in the single trial limit. *Curr. Opin*. Neurobiol. 70, 193–205. 10.1016/j.conb.2021.10.008 34861596 PMC13104381

[B132] Willumsen A. Midtgaard J. Jespersen B. Hansen C. K. K. Lam S. N. Hansen S.. (2022). Local networks from different parts of the human cerebral cortex generate and share the same population dynamic. Cereb. Cortex Commun. 3, 1–19. 10.1093/texcom/tgac040 36530950 PMC9753090

[B133] Wohrer A. Humphries M. D. Machens C. K. (2013). Population-wide distributions of neural activity during perceptual decision-making. Prog. Neurobiol. 103, 156–193. 10.1016/j.pneurobio.2012.09.004 23123501 PMC5985929

[B134] Wu H. Williams J. Nathans J. (2014). Complete morphologies of basal forebrain cholinergic neurons in the mouse. eLife 3:e02444. 10.7554/elife.0244 24894464 PMC4038840

[B135] Xu W. Huang X. Takagaki K. Wu J.-Y. (2007). Compression and reflection of visually evoked cortical waves. Neuron 55, 119–129. 10.1016/j.neuron.2007.06.016 17610821 PMC1988694

[B136] Yap M. H. W. Grabowska M. J. Rohrscheib C. Jeans R. Troup M. Paulk A. C.. (2017). Oscillatory brain activity in spontaneous and induced sleep stages in flies. Nat. Commun. 8, 1815. 10.1038/s41467-017-02024-y 29180766 PMC5704022

[B137] Zhu M. H. Jang J. Milosevich M. M. Antic S. D. (2021). Population imaging discrepancies between a genetically-encoded calcium indicator (GECI) versus a genetically-encoded voltage indicator (GEVI). Sci. Rep. 11, 5295. 10.1038/s41598-021-84651-6 33674659 PMC7935943

